# The Drosophila blood-brain barrier: development and function of a glial endothelium

**DOI:** 10.3389/fnins.2014.00365

**Published:** 2014-11-14

**Authors:** Stefanie Limmer, Astrid Weiler, Anne Volkenhoff, Felix Babatz, Christian Klämbt

**Affiliations:** Institut für Neuro- und Verhaltensbiologie, Universität MünsterMünster, Germany

**Keywords:** Drosophila, glia, blood-brain barrier, septate junction formation, transmembrane transporter, astrocyte-neuron lactate shuttle hypothesis

## Abstract

The efficacy of neuronal function requires a well-balanced extracellular ion homeostasis and a steady supply with nutrients and metabolites. Therefore, all organisms equipped with a complex nervous system developed a so-called blood-brain barrier, protecting it from an uncontrolled entry of solutes, metabolites or pathogens. In higher vertebrates, this diffusion barrier is established by polarized endothelial cells that form extensive tight junctions, whereas in lower vertebrates and invertebrates the blood-brain barrier is exclusively formed by glial cells. Here, we review the development and function of the glial blood-brain barrier of *Drosophila melanogaster*. In the Drosophila nervous system, at least seven morphologically distinct glial cell classes can be distinguished. Two of these glial classes form the blood-brain barrier. Perineurial glial cells participate in nutrient uptake and establish a first diffusion barrier. The subperineurial glial (SPG) cells form septate junctions, which block paracellular diffusion and thus seal the nervous system from the hemolymph. We summarize the molecular basis of septate junction formation and address the different transport systems expressed by the blood-brain barrier forming glial cells.

## Introduction

**Figure 1:**
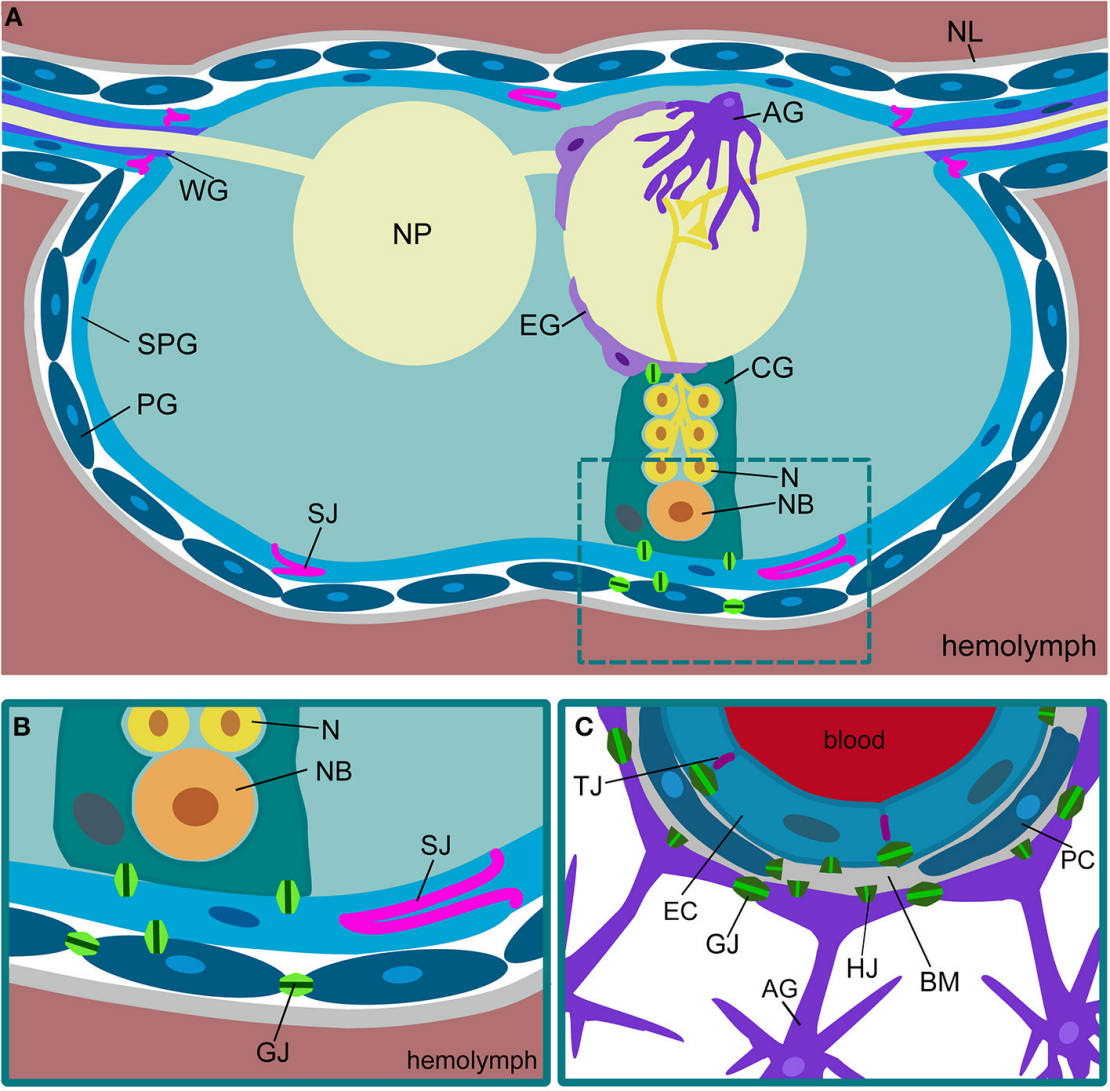
**Comparison of Drosophila and mammalian blood-brain barriers. (A)** Schematic view of a cross-section of a Drosophila ventral nerve cord. The nervous system is covered by a sheath of extracellular matrix, called neural lamella (NL). The outermost glial layer consists of perineurial glial cells (PG). The subperineurial glia (SPG) forms pleated septate junctions (SJ) and blocks paracellular transport. Neurons (N) project into the neuropil (NP). Neuronal cell bodies and neuroblasts (NB) are surrounded by cortex glia (CG). The neuropil is covered by ensheathing glia (EG). Astrocytes (AG) invade the neuropil. In the peripheral nerves, wrapping glia (WG) ensheath axons. **(B)** In Drosophila, the blood-brain barrier is built by perineurial and subperineurial glia. The latter form septate junctions (SJ) to prevent paracellular diffusion. The different glial cells are connected via gap junctions (GJ). **(C)** The mammalian blood-brain barrier is built by endothelial cells (EC) that form tight junctions (TJ) to prevent paracellular diffusion. The endothelium is in close contact with pericytes (PC). Both are surrounded by the basal membrane (BM). Gap junctions (GJ) can be found between the endothelial cells and between the astrocytes (AG). Gap junction hemichannels (HJ) can be found in all the cell types.

In all animals, an efficient separation of metabolic and ionic balance between nervous system and circulation is necessary. This in consequence led to the evolution of the so-called blood-brain barrier (Abbott et al., 2006). Vertebrates are characterized by a highly vascularized nervous system, while the insect nervous system floats in the hemolymph, which circulates through the body by the action of a primitive heart (Figures 1A–C). In the mammalian nervous system, the blood-brain barrier is established by an interplay of polarized endothelial cells and pericytes that leads to the formation of endothelial tight junctions (Armulik et al., 2010, 2011; Daneman et al., 2010b). These tight junctions prevent uncontrolled paracellular leakage of solutes into the brain. In more primitive vertebrates such as in elasmobranch fish (sharks, skates, and rays), but also in some bony fish (sturgeon), the blood-brain barrier is formed by perivascular astrocytes. These glial cells form interdigitating lamellae but do not establish tight junctions (Bundgaard and Abbott, 2008). A morphologically similar blood-brain barrier is found in insects. Here, only the outer surface of the nervous system, which is formed exclusively by glial cells, contacts the hemolymph. Although this glial barrier appears to be related to the evolutionary ancestral form of the blood-brain barrier, Drosophila has only recently emerged as a genetic model to study blood-brain barrier biology (Carlson et al., 2000; Abbott et al., 2006). Here, we summarize what is currently known on the organization and the physiological properties of the Drosophila blood-brain barrier.

Why is Drosophila a good system to study blood-brain barrier properties? One of the advantages is that the nervous system is small and almost all cells are known. In addition, geneticists established rich resource and tool kits, which allow the easy manipulation of individual cells at any time of development (Dietzl et al., 2007; Venken et al., 2011; Jenett et al., 2012; Li et al., 2014). The basis of neuronal transmission is identical in flies and man and, moreover, even complex behavioral aspects appear to be controlled by similar mechanisms (Davis, 2011; Anholt and Mackay, 2012). Thus, based on the overwhelming wealth of data documenting the evolutionary conservation of central biological processes, one can expect that work on the Drosophila blood-brain barrier, which correctly should be termed hemolymph-brain barrier, might provide further insights into the general biology of this essential boundary.

## The nervous system of drosophila

Drosophila is a holometabolic insect. Following 1 day of embryogenesis, three larval stages spread over the next 4 days. During the subsequent pupal stage, which covers another 5 days, metamorphosis takes place and the imago, the adult fly, emerges. Accordingly, the nervous system of Drosophila develops in two phases.

The larval nervous system originates from stem cells called neuroblasts that delaminate in five waves shortly after gastrulation into the interior of the embryo (Campos-Ortega and Hartenstein, 1997). Analysis of the nervous system is simplified by the fact that the thoracic and abdominal segments are mostly alike and thus, a large part of the nervous system called ventral nerve cord represents an array of repeated and almost identical neuromeric units. Currently, the identity and the lineage of all neuroblasts are known and by the end of embryogenesis about 650 neurons and 65 glial cells are found in each neuromer of the ventral nerve cord (Broadus et al., 1995; Landgraf et al., 1997; Schmid et al., 1999; Wheeler et al., 2006; Beckervordersandforth et al., 2008; Rickert et al., 2011). The brain lobes of the Drosophila larvae originate from somewhat less well-defined head neuroblasts (Urbach and Technau, 2003). The neuronal circuits established by the many neurons in the larval brain are currently being deciphered by analyzing serial TEM sections of a larval brain (Cardona et al., 2010). Thus, it can be anticipated that within the next years the complete anatomical building plan of the larval nervous system is known.

The adult nervous system, including the elaborate compound eyes, develops during early pupal stages. Neuroblast proliferation is reactivated at the end of the larval stage to generate a large number of neurons, particularly in the two brain lobes. When fully developed, the fly central nervous system harbors about 30,000 neurons (Lovick et al., 2013). Upon GFP labeling of neurons or glial cells and subsequent FACS sorting we counted about 25,000 neuronal and 10,000 glial cells per adult brain (Limmer et al., unpublished).

## Drosophila glial cells

As in primitive vertebrates, the Drosophila blood-brain barrier is formed by glial cells (Stork et al., 2008). The fly nervous system harbors seven morphologically and molecularly distinct glial subtypes, namely midline glia, perineurial glia, subperineurial glia (SPG), cortex glia, ensheathing glia, astrocytes, and wrapping glia (Figures 1A,B) (Ito et al., 1995; Pereanu et al., 2005; Silies et al., 2007; Awasaki et al., 2008; Stork et al., 2008, 2012, 2014; Hartenstein, 2011). The entire nervous system is covered by a layer of perineurial glial cells. These cells participate in blood-brain barrier function although their exact contribution is currently unknown. The subjacent glial cell layer is represented by the SPG cells. These flat and interdigitating cells form elaborate septate junctions, which prevent paracellular diffusion (Bainton et al., 2005; Stork et al., 2008; Mayer et al., 2009). The cortex glial cells engulf neuronal stem cells and their progeny and most likely exert some nutritional functions. The ensheathing glial cells form a sheath around the neuropil area, which lacks cell bodies and harbors only axons and dendrites. Astrocytes invade the neuropil to modulate synaptic transmission as it is known from their vertebrate homologs (Awasaki et al., 2008; Stork et al., 2014). The wrapping glia is mostly found in the peripheral nervous system where these cells engulf individual axons (Pereanu et al., 2005; Awasaki et al., 2008; Franzdóttir et al., 2009).

All glial subtypes are generated in the Drosophila embryo and emerge from stem cells programmed by the expression of the gene *glial cells missing* (*gcm*) (Hosoya et al., 1995; Jones et al., 1995; Vincent et al., 1996). The presence of the Gcm transcription factor specifies glial identity except for the midline glia, which requires the activity of the master regulator gene *single minded* (Crews et al., 1988). Gcm subsequently activates a cascade of transcription factors. The transcription factor Pointed promotes glial differentiation and the Zn-finger protein Tramtrack inhibits neuronal differentiation in glial cells (Klaes et al., 1994; Giesen et al., 1997). Together with other transcriptional regulators such as Prospero, Distal-less and Deadringer, these factors most likely are involved in specifying the glial subtype identity (Shandala et al., 2003; Thomas and van Meyel, 2007; Schmidt et al., 2011). The relevant transcriptional regulators that specify blood-brain barrier identity are currently unknown.

## The drosophila blood-brain barrier

The establishment of the Drosophila blood-brain barrier occurs at the end of embryogenesis and requires the SPG cells. Only 16 of these cells are formed in every neuromere and four additional SPG cells are generated along every segmental nerve (Beckervordersandforth et al., 2008; von Hilchen et al., 2013). During larval stages, when the animal grows in size by a factor of 100, and likewise during metamorphosis, no additional SPG cells are formed but the blood-brain barrier remains intact (Awasaki et al., 2008; Stork et al., 2008; Unhavaithaya and Orr-Weaver, 2012). Thus, the few SPG cells generated during embryonic stages must grow enormously in size during development, and at the same time they have to maintain their elaborate junctional contacts that prevent paracellular diffusion (see below).

Once the SPG cells are born at about mid-embryogenesis, they form numerous filopodia-like processes and eventually spread to touch their neighbors at the end of embryogenesis (Schwabe et al., 2005). The SPG cells now establish a contiguous, very flat, endothelial-like sheet that covers the entire nervous system and the cell contact zones interdigitate extensively. Interestingly, in cuttlefish as well as in sturgeon, the glial blood-brain barrier also involves highly overlapping glial lamellae (Lane and Abbott, 1992; Bundgaard and Abbott, 2008). In Drosophila, glial cells of the blood-brain barrier in addition form extensive septate junctions that further restrict the paracellular diffusion between different glial cells (Carlson et al., 2000; Schwabe et al., 2005; Stork et al., 2008). The first experimental confirmation of the physiological relevance of septate junctions for blood-brain barrier integrity was provided by the genetic analysis of the septate junction component NeurexinIV. In the absence of this protein, which is homologous to the Caspr protein found in septate-like junctions in vertebrate paranodes, the blood-brain barrier is permissive to even large molecules like dextran and in consequence, the high potassium content of the hemolymph can spread into the nervous system where it blocks any neuronal activity (Baumgartner et al., 1996).

The SPG cells are very large: a single SPG can cover the size of one half of the eye imaginal disc thus covering an area equivalent to about 10,000 epithelial cells (Silies et al., 2007). In order to achieve this enormous cell growth, the SPG cells undergo polyploidization (Unhavaithaya and Orr-Weaver, 2012). All SPG cells form a polarized endothelium that in most areas does not even reach a thickness of 1 μm. The thin nature of these barrier-forming cells has hindered detailed electron microscopic studies for a long time. Only the ability to label individual cells with GFP using subperineurial specific Gal4 driver strains such as *gliotactinGal4* or *moodyGal4* has allowed the description of the intricate morphology of these cells (Schwabe et al., 2005; Silies et al., 2007; Stork et al., 2008; Hatan et al., 2011; Unhavaithaya and Orr-Weaver, 2012). Currently, only few molecular markers for the blood-brain barrier are available. The multidrug resistance protein Mdr65 was shown to reside in the apical domain of the SPG cell, whereas the GPCR Moody is found at the basal portion of the cell (DeSalvo et al., 2011).

## Formation of septate junctions

**Figure 2:**
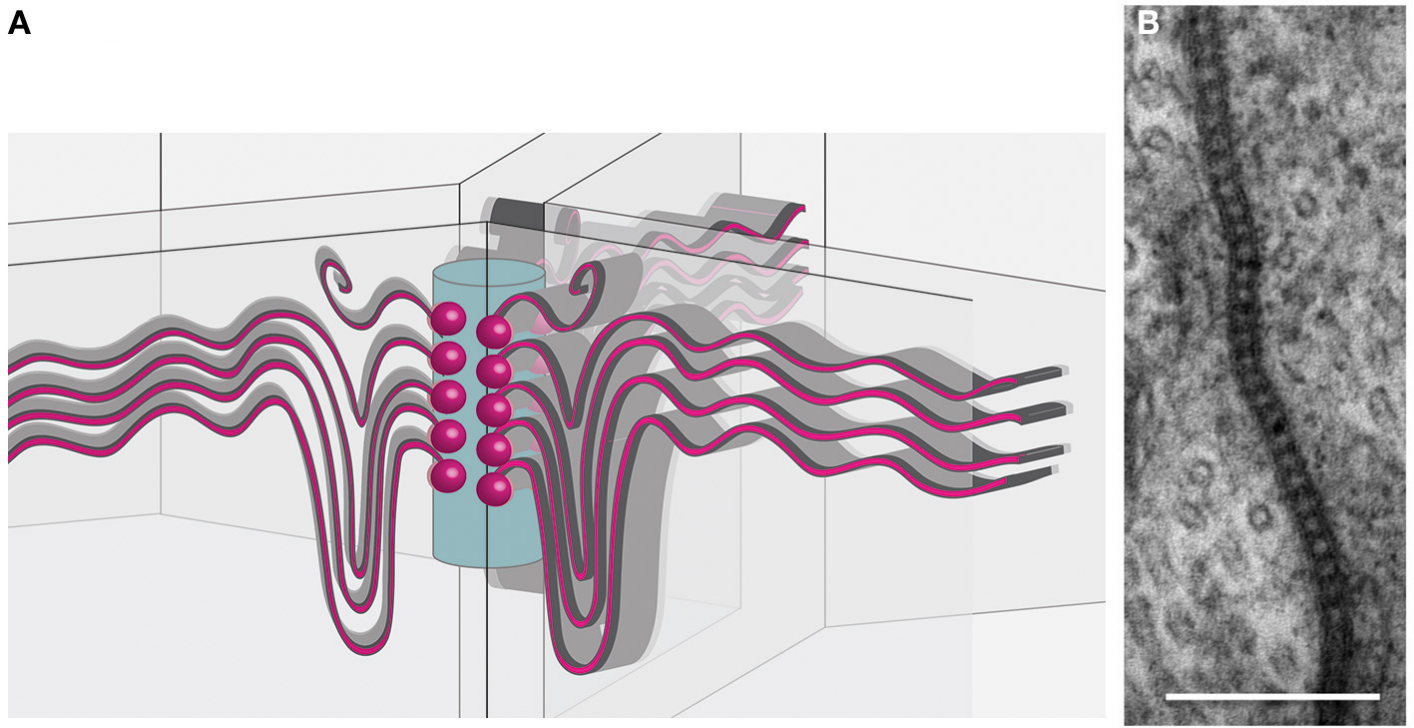
**Organization of tricellular junctions. (A)** Schematic view of septate junctions at tricellular contacts according to the tricellular plug model (Graf et al., 1982; Noirot-Timothée et al., 1982; Schulte et al., 2003). Septate junctions (red sinuous lines) span the membranes of two adjacent cells. At a central core (blue cylinder), emanating from transmembrane proteins (red balls), the septate junctions extend. **(B)** Transmission electron microscopic image of pleated septate junctions between two SPG cells.

The most characteristic feature of the SPG is the formation of extensive septate junctions. This type of cell-cell junction has been particularly well studied in ectodermal cells, such as tracheal cells (Tepass and Hartenstein, 1994). Septate junctions are a complex crystalline array of comb-like structures built by a bewildering number of different proteins that connect individual cells, as revealed by freeze-fracture studies (Figure 2) (Lane and Swales, 1979; Lane, 1991). The cell-cell distance in these junctions is about 20 nm and thus a bit larger than in tight junctions that seal the brain endothelial cells in mammals (Farquhar and Palade, 1963, 1965). Many membrane-associated proteins are known to be involved in septate junction formation (Table 1). The core group of septate junction proteins contains the cation pump ATPalpha, the claudin family members Megatrachea and Sinous, the Ig-domain protein Neuroglian, the potassium pump subunit Nervana2, the Caspr homolog NeurexinIV and the two cytoplasmic proteins Coracle and Varicose (Oshima and Fehon, 2011, see also references in Table 1). These proteins recruit a large number of additional membrane proteins that together build the septate junctions (Table 1). Loss of most of these proteins results in the disruption of septate junctions.

**Table 1:** **Septate junction proteins**.

**Gene**	**Vertebrate homolog**	**Structural domains**	**Function**	**References**
*ATP*α	NA^+^/K^+^ ATPase α-subunit		Ion pump ATPase	Genova and Fehon, 2003
*nervana 2*	NA^+^/K^+^ ATPase β-subunit		Ion pump ATPase	Genova and Fehon, 2003
*boudin*		Ly-6, GPI	Secreted, non-autonomous	Hijazi et al., 2009
*coiled*		Ly-6, GPI	Homophilic adhesion, symmetrical expression of adjacent cells necessary	Nilton et al., 2010; Syed et al., 2011
*crimpled*		Ly-6, GPI		Nilton et al., 2010
*crooked*		Ly-6, GPI		Nilton et al., 2010
*retroactive*		Ly-6	Chitin cable formation	Moussian et al., 2006
*Contactin*	Contactin	Ig, FnIII, GPI	Cell-adhesion	Faivre-Sarrailh et al., 2004
*Fasciclin III*		Ig	Cell-adhesion	Narasimha et al., 2008
*Lachesin*		Ig, GPI	Cell-adhesion	Llimargas et al., 2004
*Neuroglian*	Neurofascin 155	Ig, FnIII	Cell-adhesion	Genova and Fehon, 2003
*Neurexin IV*	Caspr/Paranodin	Laminin G and EGF domains	Cell-adhesion	Baumgartner et al., 1996
*coracle*	Protein 4.1	FERM	Linker protein	Fehon et al., 1994
*kune-kune*	Claudin	PDZ-domain	Cell-adhesion	Nelson et al., 2010
*megatrachea*	Claudin	PDZ-domain	Cell-adhesion	Jaspers et al., 2012
*sinuous*	Claudin	PDZ-domain	Cell-adhesion	Wu and Beitel, 2004
*discs large*	hDLG/Sap97	MAGUK, SH3, PDZ, guanylate kinase domain	Linker protein	Woods et al., 1996
*scribble*	hscrib1	PDZ-Domain, LRR	Linker protein	Bilder and Perrimon, 2000
*Gliotactin*	Neuroligin3	Noncatalytic cholinesterase like molecule	Cell-adhesion	Auld et al., 1995
*Macroglobulin complement-related*	α2M	Thioester protein (TEP) family		Bätz et al., 2014; Hall et al., 2014
*Melanotransferrin*	MTF	Iron binding, GPI	Iron-binding, endocytosis	Tiklová et al., 2010
*varicose*	Pals2	MAGUK	Linker protein	Wu et al., 2007
*wunen*		Lipid phosphat phosphatase		Ile et al., 2012
*yurt*				Laprise et al., 2009

*The known Drosophila septate junction proteins are listed. Structural domains and the predicted molecular functions are indicated. Ly-6, lymphocyte antigen-6; GPI, glycosyl phosphatidylinositol; Ig, immunoglobuline; FnIII, fibronectin type III; EGF, epidermal growth factor; MAGUK, membrane-associated guanylate kinases; SH3, SRC homology 3; LRR, leucine-rich repeat*.

Septate junction structure becomes even more elaborate at tricellular junctions. Here, septate junction strands of three cells meet and appear to be linked to a central core in the extracellular space between three neighboring cells (Figure 2). The tricellular junctions are among the first junctional complexes to differentiate and are characterized by several specific proteins such as Gliotactin or Macroglobin (Auld et al., 1995; Genova and Fehon, 2003; Schulte et al., 2003; Padash-Barmchi et al., 2010; Bätz et al., 2014; Furuse et al., 2014; Oda et al., 2014). Possibly septate junction formation initiates from these positions to match the stretch growth of the SPG cells during larval development (Figure 2). The extent of septate junction formation is in part controlled by the G protein-coupled receptor Moody (Bainton et al., 2005; Schwabe et al., 2005). The Moody protein is continuously required for septate junction formation and a temporal lack of *moody* function, evoked by conditional RNA interference, results in a transient opening of the blood-brain barrier (Bainton et al., 2005). It is important to note that Moody, like possibly other developmentally required proteins, may affect both, development and the physiology of the blood-brain barrier. Indeed some effects of Moody on barrier function, such as increased cocaine sensitivity, can be seen under experimental conditions (Bainton et al., 2005). However, in the normal life of a fly no serious defects are observed when *moody* function is lacking. It was noted by Schwabe et al. (2005) that *moody* mutations are lethal, but it was also mentioned that few homozygous females and hemizygous males survive to adulthood. Interestingly, homozygous *moody* flies can be easily kept as a living stock, suggesting that blood-brain barrier physiology can be efficiently regulated by additional pathways.

How Moody signaling regulates septate junction dynamics remains unclear. *moody* null mutants show normal septate junction morphology but a somewhat reduced junctional length (Schwabe et al., 2005). In addition, Moody was shown to affect the formation of actin-rich structures along the lateral borders of the SPG cells, which also contributes to the effects on blood-brain barrier integrity (Hatan et al., 2011).

In conclusion, SPG cells are intimately interconnected by septate junctions. They set up a tight seal around the nervous system, such that metabolite import into the brain, and the exit of waste products and xenobiotics out of the nervous system can be tightly controlled.

## Transport of metabolites across the blood-brain barrier

A classical function of the blood-brain barrier is to control transport of ions and metabolites. Once the septate junctions are formed between individual SPG cells, paracellular diffusion is blocked. This, in consequence, calls for efficient and highly active transport mechanisms that shuttle all required metabolites into the brain. The relevant transporters are mostly defined by bioinformatic criteria and their expression has been globally addressed by tissue specific transcriptomics and is summarized in FlyAtlas (Chintapalli et al., 2013).

Metabolite transport across the SPG cell layer not only requires an efficient uptake mechanism into the SPG cells, but also an efficient secretion of metabolites into the nervous system. This aspect may not be confined to the blood-brain barrier, since glial cells in general and in particular SPG cells establish extensive gap junctions, which is similarly observed in endothelial cells (Figure 1) (Lane and Swales, 1979; Lane, 1991; Holcroft et al., 2013; Gaete et al., 2014). Thus, once metabolites have entered the blood-brain barrier forming cells, they might be easily distributed throughout the whole nervous system via these intercellular connections. Gap junctions are a hallmark of invertebrate glia and most glial cells express several Drosophila innexins, which constitute the functional connexin homologs of invertebrates (Holcroft et al., 2013). The direct coupling of glial cells by gap junctions is also reflected by glial Ca^2+^ waves, which can be seen in the blood-brain barrier, the cortex and the astrocyte glial population (Melom and Littleton, 2013; Spéder and Brand, 2014; Stork et al., 2014).

In addition to the transport of metabolites into the brain, waste products and xenobiotics need to be shuttled out of the brain. In vertebrates, chemoprotection is mediated by ATP-binding cassette (ABC) transporters such as the Multidrug-resistance protein 1 (Mdr1). Quite similar, *mdr65* performs related functions in the Drosophila nervous system (Mayer et al., 2009). In line with this, Mdr65 levels are elevated upon exposure to insecticides (Dermauw and Van Leeuwen, 2014). Mdr65 belongs to a large class of transporters with 56 members in Drosophila of which several are expressed in the nervous system (Dermauw and Van Leeuwen, 2014). Their function, however, is largely unknown and will not be further discussed here.

Neural cells need to take up specific lipids from the hemolymph, which feeds into specific anabolic pathways (Palm et al., 2012) or is directly used as energy source during ß-oxidation (Palanker et al., 2009). In the following we will focus on metabolite import across the blood-brain barrier and review the main classes of the relevant transport systems. Due to space constrains, however, we will neglect lipid metabolism. We anticipate that the wealth of tools available in Drosophila will promote a deeper understanding of the physiological roles of this important barrier.

## Water homeostasis

Mechanisms for controlling influx and efflux of water are essential for osmotic control of fluids in the nervous system. Water can enter the nervous system through aquaporins, small membrane-spanning proteins that form channels to facilitate water flow along the osmotic gradient. 14 aquaporins are known in humans and in particular aquaporin-4 (AQP4) has been linked to blood-brain barrier function. AQP4 localizes to astrocyte endfeet at the CSF-CNS and blood-CNS barriers and is crucially required for the regulation of water homeostasis and the definition of the extracellular space in the CNS (Nagelhus and Ottersen, 2013; Papadopoulos and Verkman, 2013).

The Drosophila genome harbors eight aquaporin-encoding genes (FlyBase, CV: water transmembrane transporter activity, Table 2). The DRIP protein has the highest sequence similarity to vertebrate AQP4 but shows no prominent CNS expression (Chintapalli et al., 2013). *big brain* (*bib*) was initially identified as a neurogenic gene and encodes a protein related to aquaporins (Rao et al., 1990). Although Bib has water transport properties, this function is still controversial (Tatsumi et al., 2009). The Drosophila aquaporin most prominently expressed in the CNS is CG7777 (Chintapalli et al., 2013), but currently no functional analysis has been performed.

**Table 2:** **Drosophila Aquaporins**.

**Gene**	**CG number**	**Human homolog**	**CNS expression**			**References**
			**FlyAtlas**	**BDGP**	**Literature**	
*drip*	*CG9023*	AQP1, AQP4	–	–	No	Kaufmann et al., 2005
*big brain*	*CG4722*	AQP4	Yes	Yes	Yes	Rao et al., 1990
*aquaporin*	*CG12251*	AQP12	No	No	–	
*CG7777*	*CG7777*	AQP1	Yes	No	Yes	Kaufmann et al., 2005
*CG17664*	*CG17664*	MIWC, AQP4	No	–	No	Kaufmann et al., 2005
*CG17662*	*CG17662*	MIWC, AQP4	No	–	–	
*CG4019*	*CG4019*	MIWC, AQP4	Low	No	No	Kaufmann et al., 2005
*CG5398*	*CG5398*	AQP2	No	–	No	Kaufmann et al., 2005

*All predicted Drosophila aquaporins (FlyBase, CV: water transmembrane transporter activity) are listed. Information about the CNS expression of each gene were obtained from FlyAtlas, the Berkely Drosophila Genome Project (BDGP), and the literature shown. AQP, aquaporin; MIWC, mercurial-insensitive water channel*.

## Ion homeostasis

The hemolymph is an ion rich fluid (Table 3). In particular the high potassium concentration, which is characteristic for the invertebrate hemolymph, would be problematic for normal neuronal function. In the mammalian blood, potassium levels are only around 1–5 mM and thus almost a factor of 10 lower compared to the Drosophila hemolymph. The blood-brain barrier prevents any uncontrolled influx of ions into the nervous system. In consequence, the potassium concentration in the Drosophila brain fluid is about 5 mM or less (Armstrong et al., 2012).

**Table 3:** **Ion concentration in the hemolymph**.

**[Na^+^] [mM]**	**[K^+^] [mM]**	**[Cl^−^] [mM]**	**[Mg^2+^] [mM]**	**[Ca^2+^] [mM]**	**Remark/method**	**References**
**THIRD INSTAR LARVAL**						
52 ± 1	36 ± 1	30 ± 1	–	–	Photometry and chemical methods	Croghan and Lockwood, 1960
56.5	40.2	42.2	41.6	15.9	Physical and chemical methods	Begg and Cruickshank, 1962
~45	~23	–	–	–	Ion-selective microelectrodes	Naikkhwah and O'Donnell, 2011
**ADULT**						
70 – 110	26				Ion-selective microelectrodes	Naikkhwah and O'Donnell, 2011
123.7 ± 9.2	28.4 ± 2.4				Ion-selective microelectrodes	MacMillan and Hughson, 2014

*Drosophila third instar larval and adult hemolymph concentrations of selected ions are listed. The method of concentration determination is indicated*.

The Drosophila genome harbors a large number of proteins linked to potassium influx or export (Chintapalli et al., 2013). However, only in few cases, their relevance for regulated ion transport across the blood-brain barrier has been demonstrated. One example is the Fray kinase and its target Ncc69, the homolog of the human SLC12 Na^+^/K^+^/Cl^−^ cotransporter (Leiserson et al., 2000, 2011). Ncc69 is expressed in SPG cells and *Ncc69* mutant larvae develop a peripheral neuropathy with fluid accumulations between glia and axons (Leiserson et al., 2011). The effect of Ncc69 on CNS physiology is not reported. Several other ion transport proteins of the SLC12 family are also expressed in the Drosophila brain (Sun et al., 2010) but their possible glial functions are unexplored.

In a genetic screen for temperature-sensitive conditional seizure mutants the glial-specific Na^+^/Ca^2+^, K^+^ exchanger Zydeco was identified. Zydeco is mostly expressed by the cortex glia and not by the blood-brain barrier glia (Guan et al., 2005; Melom and Littleton, 2013). In addition to channels, active ion pumps such as the Na^+^/K^+^ ATPase are involved in regulating ion homeostasis in the brain. The two Drosophila nervana genes (*nrv1* and *nrv2*) encode beta subunits of the Na^+^/K^+^ ATPase. Nrv2 and the ATPase alpha are expressed in the SPG, where they are localized to septate junctions. Hypomorphic mutants for the ATPase alpha subunit show a bang and ouabain sensitivity (Schubiger et al., 1994). Septate junction formation, however, does not depend on ATPase function (Genova and Fehon, 2003; Paul et al., 2007).

## Uptake of amino acids into the nervous system

Amino acids are not only required for protein synthesis, but particular amino acids or their direct derivatives are also used as neurotransmitters. Cells are able to generate most amino acids from intermediates of the citric acid cycle. However, some amino acids cannot be produced and thus their uptake and transport is essential. In Drosophila, 10 proteinogenic L-amino acids are essential: tryptophan, phenylalanine, leucine, histidine, valine, isoleucine, lysine, methionine, arginine, and threonine (Boudko, 2012). Therefore, specific transport systems for these amino acids must be expressed by the SPG cells.

In vertebrates, nine distinct amino acid transport systems have been reported to be present at the brain capillary endothelium (Smith, 2000): the X-system is a high affinity, sodium-independent transport system for anionic amino acids, the L-system transports large neutral amino acids, the A-and ASC-systems transport small neutral amino acids, the Y^+^-system is a sodium-independent cationic amino acid transport system, the B^o+^-system represents a high affinity transport system and transports both neutral and basic amino acids and the ß-system is a low capacity, sodium-dependent transporter for taurine and ß-alanine. The N-system mediates the sodium-dependent transport of L-glutamine, L-histidine and L-asparagine and the T-system transports thyroid hormones.

Two well-known X-system transporters are EAAT1 and EAAT2, which in mammals are expressed by astrocytes (Rothstein et al., 1994). In Drosophila, both proteins have been described, but are expressed in glial cells other than the blood-brain barrier cells (Soustelle et al., 2002; Freeman et al., 2003; Stacey et al., 2010). In total, 51 Drosophila genes are annotated to have an “organic acid transmembrane transporter activity” or as “amino acid transporter” (Table 4). For a few of these proteins expression in CNS glial cells has been noted (Soustelle et al., 2002; Besson et al., 2005; Augustin et al., 2007; Grosjean et al., 2008; Featherstone, 2011). In some cases, expression in the blood-brain barrier was found (e.g., CG15088, Thimgan et al., 2006) but functional analysis is lacking so far.

**Table 4:** **Drosophila amino acid transporters**.

**Gene**	**CG**	**Human homolog**	**CNS expression**			**Function**	**References**
			**FlyAtlas**	**BDGP**	**Literature**		
	*CG13248*	SLC7	Yes	Yes	Yes	Cationic AA transporter; putative arginine transporter	Romero-Calderón and Krantz, 2006; Park et al., 2011
	*CG5535*	SLC7	Yes		No	Cationic AA transporter	Romero-Calderón and Krantz, 2006
	*CG12531*	SLC7	Yes			Cationic AA transporter	Romero-Calderón and Krantz, 2006
	*CG7255*	SLC7	No		No	Cationic AA transporter	Romero-Calderón and Krantz, 2006
*slimfast*	CG11128	SLC7	No			Cationic AA transporter	
*JhI-21*	*CG12317*	SLC7	Yes			L-AA transporter; lysine and other neutral AA transport light chain of heterodimeric transporter	Freeman et al., 2003; Reynolds et al., 2009
*pathetic*	*CG3424*	SLC36	Yes	Yes	Yes	AA transporter; alanine, glycine transporter	Goberdhan et al., 2005
*minidiscs*	*CG3297*	SLC7	Yes	Yes		AA transporter	Reynolds et al., 2009
*Nutrient Amino Acid transporter 1*	*CG3252*	SLC6	No		No/yes	K^+^/AA symporter; L-/D-amino acid transporter Neutral AA transporter	Thimgan et al., 2006; Miller et al., 2008; Romero-Calderón et al., 2008
	*CG17119*	CTNS	Yes			AA transporter; lysosomal L-cysteine transporter	Romero-Calderón and Krantz, 2006
	*CG1139*	SLC36	No	No		AA transporter; cysteine, alanine, glycine transport	Goberdhan et al., 2005; Romero-Calderón and Krantz, 2006
	*CG1607*	SLC7	Yes			AA transporter	Romero-Calderón and Krantz, 2006
	*CG13384*	SlC36	Yes			AA transporter	Romero-Calderón and Krantz, 2006
	*CG13743*	SLC38	Yes			AA transporter	Romero-Calderón and Krantz, 2006
	*CG7708*	SLC5	Yes	Yes		AA transporter; Proline/Na^+^ symporter; Choline transporter	Romero-Calderón and Krantz, 2006
	*CG7888*	SLC36	Yes			AA transporter	Romero-Calderón and Krantz, 2006
	*CG9413*	SLC7	Yes			AA transporter	Romero-Calderón and Krantz, 2006
*List*	*CG15088*	SLC6	Yes		Yes	K^+^/AA symporter; neuro-transmitter/Na^+^ symporter	Romero-Calderón and Krantz, 2006; Thimgan et al., 2006; Kasuya et al., 2009
	*CG30394*	SLC38	Yes			AA transporter	Romero-Calderón and Krantz, 2006
*tadr*	*CG9264*	SLC7	Yes			AA transporter	
	*CG4991*	SLC36	Yes			AA transporter	Romero-Calderón and Krantz, 2006
	*CG1628*	SLC25	Yes			AA transporter; L-ornithine transporter	Romero-Calderón and Krantz, 2006
	*CG8785*	SLC36	No			AA transporter	Romero-Calderón and Krantz, 2006
	*CG12943*	SLC36	No			AA transporter	Romero-Calderón and Krantz, 2006
	*CG13646*	no	No			AA transporter	Romero-Calderón and Krantz, 2006
	*CG32079*	SLC36	No		No	AA transporter	Romero-Calderón and Krantz, 2006
	*CG32081*	SLC36	No			AA transporter	
*hoepel1*	*CG12787*	OCA2	Yes			L-tyrosine transporter	
*hoepel2*	*CG15624*	OCA2	Yes			L-tyrosine transporter	
*kazachoc*	*CG5594*	SLC12	Yes	Yes	Yes	K^+^/Cl^−^ symporter; AA transporter	Hekmat-Scafe et al., 2006; Sun et al., 2010 Filippov et al., 2003
	*CG31547*	SLC12	Yes		Yes	AA transporter; Na^+^/K^+^/Cl^−^ symporter	Filippov et al., 2003; Romero-Calderón and Krantz, 2006; Sun et al., 2010
*Ncc69*	*CG4357*	SLC12	Yes		No/yes	Na^+^/K^+^/Cl^−^ cotransporter; AA transporter	Filippov et al., 2003; Romero-Calderón and Krantz, 2006; Sun et al., 2010; Leiserson et al., 2011
	*CG12773*	SLC12	Yes		No/yes	AA transporter; Na^+^/K^+^/Cl^−^ symporter	Filippov et al., 2003; Romero-Calderón and Krantz, 2006; Sun et al., 2010
	*CG1698*	SLC6	No		No	K^+^/Cl^−^ symporter	Romero-Calderón and Krantz, 2006; Thimgan et al., 2006
*karmoisin*	*CG12286*	SLC16	Yes			Monocarboxylic acid transporter	
*Silnoon*	*CG8271*	SLC16	Yes			Secondary active monocarboxylate transporter; Butyrate, lactate transport	Jang et al., 2008
*outsiders*	*CG8062*	SLC16	No			Monocarboxylic acid transporter	
*Dietary and metabolic glutamate transporter*	*CG5304*	SLC17	Yes		No	High affinity inorganic phosphate/Na^+^ symporter; Glutamate transporter; Na^+^-independent glutamate transporter	Laridon et al., 2008; Shim et al., 2011
*Eaat 1*	*CG3747*	SLC1	Yes		Yes	Glutamate/Na^+^ symporter; L-aspartate transporter; Na^+^/Dicarboxylate symporter; L-Glutamate transporter	Besson et al., 2000; Freeman et al., 2003; Rival et al., 2004
*Eaat 2*	*CG3159*	SLC1	Yes		Yes	Glutamate/Na^+^ transporter; L-aspartate Taurine transporter; Na^+^/Dicarboxylate symporter	Besson et al., 2000, 2005, 2011; Soustelle et al., 2002 Freeman et al., 2003
*genderblind*	*CG6070*	SLC7	Yes	Yes	Yes	AA transporter Glutamate transporter	Augustin et al., 2007, Grosjean et al., 2008
*Ntl*	*CG7075*	SLC6	No		No	Neurotransmitter/Na^+^ symporter; Glycine transporter	Thimgan et al., 2006; Romero-Calderón et al., 2008
*Vesicular GABA transporter*	*CG8394*	SLC3	Yes		Yes	GABA/H^+^ symporter; AA transporter	Romero-Calderón et al., 2008; Fei et al., 2010
*Vesicular glutamate transporter*	*CG9887*	SLC17	Yes		Yes	High affinity inorganic phosphate/Na^+^ symporter; L-glutamate transporter	Daniels et al., 2004
	*CG1732*	SLC6	Yes	Yes	Yes	GABA/Na^+^ symporter	Romero-Calderón and Krantz, 2006; Thimgan et al., 2006
	*CG4476*	SLC6	Yes		No	K^+^/AA symporter; Neurotransmitter/Na^+^ symporter	Romero-Calderón and Krantz, 2006; Thimgan et al., 2006
	*CG16700*	SLC36	Yes			AA transporter; GABA/H^+^ symporter	Romero-Calderón and Krantz, 2006
	*CG5549*	SLC6	No		Yes	Glycine/Na^+^ symporter	Romero-Calderón and Krantz, 2006; Thimgan et al., 2006
	*CG8850*	SLC6	No		No	K^+^/AA symporter; Na^+^/AA symporter	Romero-Calderón and Krantz, 2006; Thimgan et al., 2006
	*CG13796*	No	No			Glycine/Na^+^ symporter	Romero-Calderón and Krantz, 2006
	*CG15279*	SLC5	No		No	Cation/Na^+^ symporter	Romero-Calderón and Krantz, 2006; Thimgan et al., 2006

*Amino acid transporters annotated in FlyBase as “organic acid transmembrane transporter activity” or as “amino acid transporter” are listed. Information about the CNS expression of each gene was obtained from FlyAtlas, the Berkely Drosophila Genome Project (BDGP), or literature. The predicted function of each protein is indicated. SLC, solute carrier; AA, amino acid; OCA2, oculocutaneous albinism II; GABA, γ-Aminobutyric acid*.

## Energy supply of the brain

The energy expenditure for normal neuronal function is enormous. The human brain requires about 20% of the total resting oxygen consumption of the entire body although it comprises only about 2% of the body mass. Likewise in flies, just the photoreceptor cells in the retina consume about 10% of the total ATP production (Laughlin et al., 1998). Therefore, an efficient transport of energy-rich nutrients such as sugars needs to be established at the blood-brain barrier.

## The astrocyte-neuron lactate shuttle hypothesis

The intense metabolic interactions between glial cells and neurons led to the astrocyte-neuron lactate shuttle (ANLS) hypothesis (Pellerin and Magistretti, 1994, 2012; Allaman et al., 2011). In the mammalian brain, the main energy source is glucose, which is shuttled into the nervous system via the Glut1 transporter. Glut1 is asymmetrically expressed in endothelial cells and is also found in astrocytes surrounding the endothelium (Leybaert, 2005). Glucose that is taken up by astrocytes is then metabolized through glycolysis to lactate or pyruvate. These small C_3_ metabolites are then released into the extracellular space to be utilized by neurons, which is supported by experimental and as well as theoretical considerations (Rouach et al., 2008; Jolivet et al., 2009; Harris et al., 2012). Enhanced neuronal activity might be linked to an increase in Glut1 expression, which would account for increased energy supply (Leybaert, 2005). This energetic coupling may be of more general relevance, since also the survival of myelinated axons depends on metabolic support by the corresponding glial cells (Fünfschilling et al., 2012; Lee et al., 2012). In invertebrates, a similar compartmentalization of energy metabolism is likely to be established as well. In the honeybee retina, glucose is exclusively taken up by glial cells and alanine, which is generated from pyruvate through transamination, seems to be shuttled to neurons to fuel the TCA cycle (Tsacopoulos et al., 1994).

## Trehalose transporters in the drosophila blood-brain barrier

Several different sugars are present in the Drosophila hemolymph (Table 5). The main carbohydrate found in the insect hemolymph is trehalose, a non-reducing disaccharide with two D-glucose units linked by an α,α-1,1-glycosidic bond. Smaller amounts of glucose and fructose are also found, but their respective concentrations appear to vary depending on the metabolic state of the animal (Blatt and Roces, 2001). Upon feeding, carbohydrates are taken up by the intestinal epithelium and glucose is secreted into the hemolymph. The glucose is then shuttled to the fat body, where trehalose is synthesized from glucose-6-phosphate and UDP-glucose by the enzyme Trehalose phosphate synthase, which is encoded by an essential gene in Drosophila (Tps1, CG4104, Chen and Haddad, 2004). Among others, trehalose is used for the maintenance of energy metabolism during fasting and non-feeding periods (Friedman, 1978; Arrese and Soulages, 2010; Chen et al., 2010).

**Table 5:** **Sugar concentration in the insect hemolymph**.

**Organism**	**Trehalose**	**Glucose**	**Fructose**	**Remark**	**References**
*A. mori, G. mellonella, T. polyphemus, P. cecropia*	2–13 mg/ml	0–2.8 mg/ml	Not tested	Based on chromatography and chemical methods	Wyatt and Kalf, 1957
Several insects	2–50 mg/ml	Generally low amounts, but *Apis mellifica* 6–32 mg/ml, *Phormia regina* 7–12.5 mg/ml	Generally low amounts, but *Apis mellifica* 2–16 mg/ml	Review on hemolymph composition in insects	Jeuniaux, 1971
*Apis mellifica*	40 mg/ml	10 mg/ml	10 mg/ml	HPLC, trehalose concentration changes depending on metabolic rate	Blatt and Roces, 2001
Drosophila larvae	60 mg/ml	50 mg/ml	Not tested	Commercial kit	Lee and Park, 2004
Drosophila larvae	13.7–17.2 mg/ml	5.4–7.2 mg/ml	Not tested	Commercial kit	Broughton et al., 2008
Drosophila larvae	6 mg/ml	1 mg/ml	Not tested	Commercial kit	Pasco and Léopold, 2012
Drosophila adults	17.2 mg/ml	1.8 mg/ml	Not tested	Commercial kit	Broughton et al., 2008

*Trehalose, glucose, and fructose concentrations in the hemolymph of different organisms and developmental stages are listed. Methods for concentration measurements are indicated*.

These observations suggest that specific sugar transporters exist, which import either trehalose or glucose into the brain. Two dedicated trehalose transporters, Tret1-1 and Tret1-2, have been described in *Drosophila melanogaster* (Kikawada et al., 2007). A comparison with other insect species shows that only one trehalose transporter gene is conserved in insects whereas the second trehalose transporter gene found in *Drosophila melanogaster* (*Tret1-2*) arose from a recent gene duplication event. Tret1-1 but not Tret1-2 is able to transport trehalose when expressed in Xenopus oocytes suggesting that the two trehalose transporters exert non-redundant functions (Kanamori et al., 2010). Trehalose transporters belong to the solute carrier 2 (SLC2) facilitated glucose transporter family with strongest homology to the human SLC2A8 protein. According to microarray data, Tret1-1 is strongly expressed in the brain. Tret1-2 is expressed only at very low levels. Its function is currently unknown (Kanamori et al., 2010). In conclusion, the Trehalose transporter is in a prime position to control the import of high-energy carbohydrates at the blood-brain barrier.

## Carbohydrate transporters in the drosophila blood-brain barrier

In total, 78 genes of Drosophila harbor a sugar transporter motif (Interpro domain search; IPR005829, Table 6). Most of the encoded proteins, however, probably will not transport sugar across the plasma membrane but, as the SLC35 member Meigo, organize intracellular trafficking of different sugars to ensure glycosylation (Sekine et al., 2013). As an alternative to trehalose, other sugars such as glucose or fructose could be taken up by the nervous system to meet the high neuronal energy demand. In vertebrates, the SLC2 family includes Glut2 and Glut5 transporters, which have been characterized as glucose and fructose transporters (Douard and Ferraris, 2008; Kellett et al., 2008; Mueckler and Thorens, 2013). No clear homolog of Glut2 and Glut5 can be identified in Drosophila, so it remains uncertain whether fructose can be imported into the nervous system. Interestingly, however, a fructose receptor (Gr43a) has been shown to be expressed on several CNS neurons (Miyamoto et al., 2012). Thus, a possible explanation for the apparent lack of Glut2 or Glut5 homologs is that fructose might be either generated in the brain or that the fructose sensing CNS neurons form dendrites that leave the CNS to detect fructose in the hemolymph.

**Table 6:** **Drosophila sugar transporters**.

**Gene**	**CG**	**Human homolog**	**CNS expression**		**Function**	**Reference**
			**FlyAtlas**	**BDGP**		
	*CG10960*	SLC2A8 (Glut8)	Yes	Yes	glucose transporter	
*Tret1-1*	*CG30035*	SLC2A8 (Glut8)	Yes		glucose transporter; trehalose transport	Kikawada et al., 2007; Kanamori et al., 2010
*Glut1*	*CG1086*	SLC2	Yes	Yes	glucose transporter	
	*CG1213*	SLC2	Yes		glucose transporter	
*sut1*	*CG8714*	SLC2	Yes		sugar/H^+^ symporter; glucose transporter	
*Glut3*	*CG3853*	SLC2	No		sugar transporter; glucose transporter	
*Tret1-2*	*CG8234*	SLC2A8 (Glut8)	No		fructose transporter; glucose transporter;	Kanamori et al., 2010
*sut2*	*CG17975*	SLC2	No		sugar/H^+^ symporter; glucose transporter	
*sut3*	*CG17976*	SLC2	No		sugar/H^+^ symporter; glucose transporter	
*sut4*	*CG1380*	SLC2	No		sugar/H^+^ symporter; glucose transporter	
	*CG1208*	SLC2	Yes		glucose transporter	
	*CG7882*	SLC2	No		glucose transporter	
	*CG8249*	SLC2	No		glucose transporter	
	*CG4797*	SLC2	Yes		glucose transporter	
	*CG8837*	SLC2	Yes		transporter activity	
	*CG15406*	SLC2	No		fructose transporter	
	*CG4607*	SLC2	Yes		substrate-specific transporter	
	*CG3285*	SLC2	No		transporter activity	
	*CG33281*	SLC2	No		monosaccaride transporter	
	*CG6484*	SLC2	No		glucose transporter	
	*CG33282*	SLC2	No		monosaccaride transporter	
	*CG15408*	SLC2	No		fructose transporter	
	*CG14605*	SLC2	No		transmembrane transporter activity	
	*CG14606*	SLC2	No		hexose transporter	
	*CG11976*	SLC2	Yes		monosaccaride transporter	
	*CG3168*	SLC22/SV2	Yes	Yes	transporter activity	Altenhein et al., 2006
	*CG6126*	SLC22	Yes	Yes	organic cation transporter	
*Orct*	*CG6331*	SLC22	Yes	Yes	organic cation transporter	
	*CG3790*	SLC22	Yes		carnitine transporter	
	*CG6356*	SLC22	Yes		secondary active organic cation transporter	
	*CG7084*	SLC22	Yes		secondary active organic cation transporter	
	*CG7442*	SLC22	Yes		secondary active organic cation transporter	
*Orct2*	*CG13610*	SLC22	Yes	Yes	organic cation transporter	
	*CG8654*	SLC22	Yes	Yes	secondary active organic cation transporter	
	*CG10486*	SLC22	No		secondary active organic cation transporter	
	*CG16727*	SLC22	No		organic cation transporter	
	*CG17751*	SLC22	No		secondary active organic cation transporter	
	*CG17752*	SLC22	No		secondary active organic cation transporter	
	*CG14855*	SLC22	Yes		secondary active organic cation transporter	
	*CG14856*	SLC22	No		secondary active organic cation transporter	
	*CG6006*	SLC22	Yes		carnitine transporter	
	*CG6231*	SLC22	Yes		secondary active organic cation transporter	
	*CG7333*	SLC22	No		secondary active organic cation transporter	
	*CG5592*	SLC22	No		secondary active organic cation transporter	
	*CG7342*	SLC22	No		secondary active organic cation transporter	
	*CG42269*	SLC22	No		secondary active organic cation transporter	
	*CG7458*	SLC22	No		secondary active organic cation transporter	
	*CG8925*	SLC22	No		carnitine transporter	
	*CG9317*	SLC22	Yes		secondary active organic cation transporter	
*Csat*	*CG2675*	SLC35	Yes		UDP-galactose transporter; sugar/H^+^ symporter	Segawa et al., 2002
*Efr*	*CG3774*	SLC35	Yes		nucleotide-sugar transporter; GDP-fuctose transporter	
*frc*	*CG3874*	SLC35	Yes		triose-phosphate transporter; pyrimidine nucleotide-sugar transporter	
*meigo*	*CG5802*	SLC35	Yes		UDP-N-acetylglucosamine transporter	
*sll*	*CG7623*	SlC35	Yes		UDP-N-acetylglucosamine transporter; 3′-phosphoadenosine 5′-phosphosulfate transporter	
*Papst2*	*CG7853*	SLC35	Yes		UDP-N-acetylglucosamine transporter; 3′-phosphoadenosine 5′-phosphosulfate transporter	
*Gfr*	*CG9620*	SLC35	Yes		GDP-fucose transporter	
	*CG33181*	SLC41	Yes		cation transporter	
	*CG11537*	HIAT1	Yes		carbohydrate transporter	
*Prp38*	*CG30342*	PRPF38	Yes		pre-mRNA-splicing factor	
	*CG7009*	FTSJ1	Yes		tRNA methyltransferase activity	
*slv*	*CG8717*	SLC50	Yes		sweet sugar transporter	
*rtet*	*CG5760*	MFSD	Yes		sugar transporter	
	*CG15096*	SLC17	Yes		high affinity inorganic phosphate/Na^+^ symporter	
	*CG30344 (CG8054)*	SLC46	Yes		transporter activity	Freeman et al., 2003
	*CG6901*	SVOP	No		transporter activity	
	*CG12783*	SV2	No		transporter activity	
	*CG30345*	SLC46	No		transporter activity	
	*CG15553*	SLC46	No		transporter activity	
	*CG14160*	–	No		transporter activity	
	*CG17929*	–	No		transporter activity	
	*CG17930*	–	No		transporter activity	
	*CG32053*	–	No		transporter activity	
	*CG32054*	–	No		transporter activity	
	*CG42825*	–	No		transporter activity	
	*CG31103*	SV2	No		transporter activity	
	*CG33233*	SV2	No		transporter activity	
	*CG4324*	SVOPL	No		organic cation transporter	
	*CG5078*	HIAT1	No		carbohydrate transporter	

*Carbohydrate transporters harboring a sugar transporter motif (Interpro domain search, IPR005829) are listed. Information about the CNS expression of each gene was obtained as indicated. The predicted function of each protein is noted. SLC, solute carrier; HIAT, hippocampus abundant transcript 1; PRPF, pre-mRNA processing factor; FTSJ1, FtsJ RNA methyltransferase homolog 1; MFSD10, major facilitator superfamily domain containing 10; SVOP, SV2 related protein homolog; SV2, synaptic vesicle glycoprotein 2; SVOPL, SVOP-like*.

Unlike in the Drosophila hemolymph, glucose is the main energy supply in the mammalian blood. Glucose is transported by Glut1 and Glut3, which are also members of the SLC2 family. For both of these facilitated glucose transporters, glucose, galactose, and mannose transport activities have been found (Uldry and Thorens, 2004). Glut1 functions in the mammalian endothelial blood-brain barrier and in astrocytes, whereas Glut3 is expressed by neurons (Leino et al., 1997). In Drosophila, Glut1 is specifically expressed in the embryonic nervous system and microarray data indicate continued expression in brain tissue (FlyBase). Future work needs to discriminate whether Glut1 is expressed in neurons or glial cells or both. In contrast, Glut3 is only expressed in imaginal discs of late larval stages and in adult testis (FlyBase). Thus, Glut1 might be responsible for glucose uptake into the nervous system. In support of this notion, loss of *glut1* function is lethal (Saito et al., 2002) and expression of *glut1* is able to improve locomotor behavior and survival of flies when mitochondrial activity is reduced in glial cells (Besson et al., 2010).

In addition, members of the SLC5A family (SLC5A1 and SLC5A2) have been shown to mediate sodium-dependent glucose uptake (Featherstone, 2011). One Drosophila SLC5A family member is CG9657, which is also expressed in glial cells (Freeman et al., 2003).

## Blood-brain barrier and hormonal function

In addition to the control of metabolism, the blood-brain barrier must also permit the entry and exit of hormones into or out of the nervous system. This is especially true for the Drosophila neuroendocrine system, which consists of neurosecretory cells (NSCs) in the brain. The Drosophila genome encodes eight insulin-like peptides (Dilps), which are the functional homologs of vertebrate insulin and insulin-like growth factors (IGFs) that affect a wide range of processes (Erion and Sehgal, 2013; Shim et al., 2013). Dilp2, 3 and 5 are expressed by 14 insulin-producing cells (IPCs) and released into the hemolymph to regulate growth, metabolism, reproduction, and life span (Nässel et al., 2013). A key trigger of Dilp release from IPCs is food intake. The associated increase in hemolymph sugar and amino acid levels is sensed by the fat body and causes release of the leptin-like peptide Unpaired 2 (Upd2), which acts on IPCs via GABAergic neurons (Rajan and Perrimon, 2012). However, how this fat body-derived signal passes the blood-brain barrier or how it is sensed by the surface glia and then further transmitted into the nervous system is not known.

An additional fat body derived signal coordinates the second wave of neurogenesis at the end of larval stages, which must be matched to the nutritional status of the animal. This still elusive signal triggers expression of Dilp6 in the SPG, which in turn activates the proliferation of larval neuroblasts (Chell and Brand, 2010; Sousa-Nunes et al., 2011). Interestingly, both, the expression and the secretion of Dilp6 from the SPG cells depend on the presence of gap junctions in these cells (Spéder and Brand, 2014).

Moreover, the blood-brain barrier influences the physiology of the animal. It is known that some hemolymph proteins modulate the mating behavior of Drosophila (Lazareva et al., 2007). Interestingly, the sex of the blood-brain barrier matters and male-specific factors of the blood-brain barrier are required for normal male courtship behavior (Hoxha et al., 2013). These aspects of blood-brain barrier function are currently not extensively studied but it appears likely, that in the near future more surprising findings will be made.

## Conclusions

Nervous system function strongly depends on a well-balanced ion and metabolite milieu. To ensure this homeostasis in the brain, the blood-brain barrier fulfills a variety of functions. It seals the brain from circulation and, in consequence, active transport systems are required for all solutes that have to be shuttled into or out of the brain. Therefore, a number of specific transporters must be expressed in the barrier forming cells. In Drosophila, as in primitive vertebrates, the blood-brain barrier is formed by glia. To date, our knowledge about essential transporters expressed in the glia is very limited. However, genomic information and transcriptomic data as already available for the mouse (Daneman et al., 2010a) will soon enable us to identify many relevant genes in Drosophila and the wealth of genetic tools will ease their analysis. One of the future challenges will be to decipher how the metabolic supply through the blood-brain barrier is matched to neuronal activity. In light of the apparently well-conserved blood-brain barrier, functional studies using Drosophila are expected to deepen our understanding of how the blood-brain barrier keeps our nervous system functional.

### Conflict of interest statement

The authors declare that the research was conducted in the absence of any commercial or financial relationships that could be construed as a potential conflict of interest.

## Abbreviations

ABC: ATP binding cassette
ATP: Adenosine triphosphate
FACS: fluorescence-activated cell sorting
GCM: Glial Cells Missing
GFP: green flourescent protein
GPCR: G protein-coupled receptor
IPC: Insulin-Producing Cell
RNA: Ribonucleic acid
SPG: subperineurial glia
TCA cycle: tricarboxylic acid cycle
UDP: Uridine diphosphate.

## References

[B1] Abbott N. J. Rönnbäck L. Hansson E. (2006). Astrocyte-endothelial interactions at the blood-brain barrier. Nat. Rev. Neurosci. 7, 41–53. 10.1038/nrn1824 16371949

[B2] Allaman I. Bélanger M. Magistretti P. J. (2011). Astrocyte-neuron metabolic relationships: for better and for worse. Trends Neurosci. 34, 76–87. 10.1016/j.tins.2010.12.001 21236501

[B3] Altenhein B. Becker A. Busold C. Beckmann B. Hoheisel J. D. Technau G. M. (2006). Expression profiling of glial genes during Drosophila embryogenesis. Dev. Biol. 296, 545–560. 10.1016/j.ydbio.2006.04.460 16762338

[B4] Anholt R. R. H. Mackay T. F. C. (2012). Genetics of aggression. Annu. Rev. Genet. 46, 145–164. 10.1146/annurev-genet-110711-155514 22934647

[B5] Armstrong G. A. B. Rodríguez E. C. Meldrum Robertson R. (2012). Cold hardening modulates K+ homeostasis in the brain of *Drosophila melanogaster* during chill coma. J. Insect Physiol. 58, 1511–1516. 10.1016/j.jinsphys.2012.09.006 23017334

[B6] Armulik A. Genové G. Betsholtz C. (2011). Pericytes: developmental, physiological, and pathological perspectives, problems, and promises. Dev. Cell 21, 193–215. 10.1016/j.devcel.2011.07.001 21839917

[B7] Armulik A. Genové G. Mäe M. Nisancioglu M. H. Wallgard E. Niaudet C.. (2010). Pericytes regulate the blood-brain barrier. Nature 468, 557–561. 10.1038/nature09522 20944627

[B8] Arrese E. L. Soulages J. L. (2010). Insect fat body: energy, metabolism, and regulation. Annu. Rev. Entomol. 55, 207–225. 10.1146/annurev-ento-112408-085356 19725772 PMC3075550

[B9] Augustin H. Grosjean Y. Chen K. Sheng Q. Featherstone D. E. (2007). Nonvesicular release of glutamate by glial xCT transporters suppresses glutamate receptor clustering *in vivo*. J. Neurosci. 27, 111–123. 10.1523/JNEUROSCI.4770-06.2007 17202478 PMC2193629

[B10] Auld V. J. Fetter R. D. Broadie K. Goodman C. S. (1995). Gliotactin, a novel transmembrane protein on peripheral glia, is required to form the blood-nerve barrier in Drosophila. Cell 81, 757–767. 10.1016/0092-8674(95)90537-5 7539719

[B11] Awasaki T. Lai S.-L. Ito K. Lee T. (2008). Organization and postembryonic development of glial cells in the adult central brain of Drosophila. J. Neurosci. 28, 13742–13753. 10.1523/JNEUROSCI.4844-08.2008 19091965 PMC6671902

[B12] Bainton R. J. Tsai L. T.-Y. Schwabe T. DeSalvo M. Gaul U. Heberlein U. (2005). moody encodes two GPCRs that regulate cocaine behaviors and blood-brain barrier permeability in Drosophila. Cell 123, 145–156. 10.1016/j.cell.2005.07.029 16213219

[B13] Bätz T. Förster D. Luschnig S. (2014). The transmembrane protein Macroglobulin complement-related is essential for septate junction formation and epithelial barrier function in Drosophila. Development 141, 899–908. 10.1242/dev.102160 24496626

[B14] Baumgartner S. Littleton J. T. Broadie K. Bhat M. A. Harbecke R. Lengyel J. A.. (1996). A Drosophila neurexin is required for septate junction and blood-nerve barrier formation and function. Cell 87, 1059–1068. 10.1016/S0092-8674(00)81800-0 8978610

[B15] Beckervordersandforth R. M. Rickert C. Altenhein B. Technau G. M. (2008). Subtypes of glial cells in the Drosophila embryonic ventral nerve cord as related to lineage and gene expression. Mech. Dev. 125, 542–557. 10.1016/j.mod.2007.12.004 18296030

[B16] Begg M. Cruickshank W. J. (1962). A partial analysis of Drosophila larval hæmolymph. Proc. R Soc. Edinb. B Biol. 68, 215–236 10.1017/S0080455X00001053

[B17] Besson M.-T. Dupont P. Fridell Y.-W. C. Liévens J.-C. (2010). Increased energy metabolism rescues glia-induced pathology in a Drosophila model of Huntington's disease. Hum. Mol. Genet. 19, 3372–3382. 10.1093/hmg/ddq249 20566711

[B18] Besson M.-T. Ré D. B. Moulin M. Birman S. (2005). High affinity transport of taurine by the Drosophila aspartate transporter dEAAT2. J. Biol. Chem. 280, 6621–6626. 10.1074/jbc.M412440200 15611131

[B19] Besson M. T. Sinakevitch I. Melon C. Iché-Torres M. Birman S. (2011). Involvement of the Drosophila taurine/aspartate transporter dEAAT2 in selective olfactory and gustatory perceptions. J. Comp. Neurol. 519, 2734–2757. 10.1002/cne.22649 21484805

[B20] Besson M. T. Soustelle L. Birman S. (2000). Selective high-affinity transport of aspartate by a Drosophila homologue of the excitatory amino-acid transporters. Curr. Biol. 10, 207–210. 10.1016/S0960-9822(00)00339-0 10704415

[B21] Bilder D. Perrimon N. (2000). Localization of apical epithelial determinants by the basolateral PDZ protein Scribble. Nature 403, 676–680. 10.1038/35001108 10688207

[B22] Blatt J. Roces F. (2001). Haemolymph sugar levels in foraging honeybees (*Apis mellifera* carnica): dependence on metabolic rate and *in vivo* measurement of maximal rates of trehalose synthesis. J. Exp. Biol. 204, 2709–2716. 11533121 10.1242/jeb.204.15.2709

[B23] Boudko D. Y. (2012). Molecular basis of essential amino acid transport from studies of insect nutrient amino acid transporters of the SLC6 family (NAT-SLC6). J. Insect Physiol. 58, 433–449. 10.1016/j.jinsphys.2011.12.018 22230793 PMC3397479

[B24] Broadus J. Skeath J. B. Spana E. P. Bossing T. Technau G. Doe C. Q. (1995). New neuroblast markers and the origin of the aCC/pCC neurons in the Drosophila central nervous system. Mech. Dev. 53, 393–402. 10.1016/0925-4773(95)00454-8 8645605

[B25] Broughton S. Alic N. Slack C. Bass T. Ikeya T. Vinti G.. (2008). Reduction of DILP2 in Drosophila triages a metabolic phenotype from lifespan revealing redundancy and compensation among DILPs. PLoS ONE 3:e3721. 10.1371/journal.pone.0003721 19005568 PMC2579582

[B26] Bundgaard M. Abbott N. J. (2008). All vertebrates started out with a glial blood-brain barrier 4-500 million years ago. Glia 56, 699–708. 10.1002/glia.20642 18338790

[B27] Campos-Ortega J. Hartenstein V. (1997). The Embryonic Development of Drosophila Melanogaster. 2nd Edn Berlin: Springer-Verlag 10.1007/978-3-662-22489-2

[B28] Cardona A. Saalfeld S. Preibisch S. Schmid B. Cheng A. Pulokas J.. (2010). An integrated micro- and macroarchitectural analysis of the Drosophila brain by computer-assisted serial section electron microscopy. PLoS Biol 8:e1000502. 10.1371/journal.pbio.1000502 20957184 PMC2950124

[B29] Carlson S. D. Juang J. L. Hilgers S. L. Garment M. B. (2000). Blood barriers of the insect. Annu. Rev. Entomol. 45, 151–174. 10.1146/annurev.ento.45.1.151 10761574

[B30] Chell J. M. Brand A. H. (2010). Nutrition-responsive glia control exit of neural stem cells from quiescence. Cell 143, 1161–1173. 10.1016/j.cell.2010.12.007 21183078 PMC3087489

[B31] Chen J. Tang B. Chen H. Yao Q. Huang X. Chen J.. (2010). Different functions of the insect soluble and membrane-bound trehalase genes in chitin biosynthesis revealed by RNA interference. PLoS ONE 5:e10133. 10.1371/journal.pone.0010133 20405036 PMC2853572

[B32] Chen Q. Haddad G. G. (2004). Role of trehalose phosphate synthase and trehalose during hypoxia: from flies to mammals. J. Exp. Biol. 207, 3125–3129. 10.1242/jeb.01133 15299033

[B33] Chintapalli V. R. Wang J. Herzyk P. Davies S. A. Dow J. A. (2013). Data-mining the FlyAtlas online resource to identify core functional motifs across transporting epithelia. BMC Genomics 14:518. 10.1186/1471-2164-14-518 23895496 PMC3734111

[B34] von Hilchen C. M. Bustos Á. E. Giangrande A. Technau G. M. Altenhein B. (2013). Predetermined embryonic glial cells form the distinct glial sheaths of the Drosophila peripheral nervous system. Development 140, 3657–3668. 10.1242/dev.093245 23903191 PMC3915570

[B35] Crews S. T. Thomas J. B. Goodman C. S. (1988). The Drosophila single-minded gene encodes a nuclear protein with sequence similarity to the per gene product. Cell 52, 143–151. 10.1016/0092-8674(88)90538-7 3345560

[B36] Croghan P. C. Lockwood A. P. M. (1960). The composition of the hemolymph of the larva of *Drosophila melanogaster*. J. Exp. Biol. 37, 339–343.

[B37] Daneman R. Zhou L. Agalliu D. Cahoy J. D. Kaushal A. Barres B. A. (2010a). The mouse blood-brain barrier transcriptome: a new resource for understanding the development and function of brain endothelial cells. PLoS ONE 5:e13741. 10.1371/journal.pone.0013741 21060791 PMC2966423

[B38] Daneman R. Zhou L. Kebede A. A. Barres B. A. (2010b). Pericytes are required for blood-brain barrier integrity during embryogenesis. Nature 468, 562–566. 10.1038/nature09513 20944625 PMC3241506

[B39] Daniels R. W. Collins C. A. Gelfand M. V. Dant J. Brooks E. S. Krantz D. E.. (2004). Increased expression of the Drosophila vesicular glutamate transporter leads to excess glutamate release and a compensatory decrease in quantal content. J. Neurosci. 24, 10466–10474. 10.1523/JNEUROSCI.3001-04.2004 15548661 PMC6730318

[B40] Davis R. L. (2011). Traces of Drosophila memory. Neuron 70, 8–19. 10.1016/j.neuron.2011.03.012 21482352 PMC3374581

[B41] Dermauw W. Van Leeuwen T. (2014). The ABC gene family in arthropods: comparative genomics and role in insecticide transport and resistance. Insect Biochem. Mol. Biol. 45, 89–110. 10.1016/j.ibmb.2013.11.001 24291285

[B42] DeSalvo M. K. Mayer N. Mayer F. Bainton R. J. (2011). Physiologic and anatomic characterization of the brain surface glia barrier of Drosophila. Glia 59, 1322–1340. 10.1002/glia.21147 21351158 PMC3130812

[B43] Dietzl G. Chen D. Schnorrer F. Su K.-C. Barinova Y. Fellner M.. (2007). A genome-wide transgenic RNAi library for conditional gene inactivation in Drosophila. Nature 448, 151–156. 10.1038/nature05954 17625558

[B44] Douard V. Ferraris R. P. (2008). Regulation of the fructose transporter GLUT5 in health and disease. Am. J. Physiol. Endocrinol. Metab. 295, E227–E237. 10.1152/ajpendo.90245.2008 18398011 PMC2652499

[B45] Erion R. Sehgal A. (2013). Regulation of insect behavior via the insulin-signaling pathway. Front. Physiol. 4:353. 10.3389/fphys.2013.00353 24348428 PMC3847551

[B46] Faivre-Sarrailh C. Banerjee S. Li J. Hortsch M. Laval M. Bhat M. A. (2004). Drosophila contactin, a homolog of vertebrate contactin, is required for septate junction organization and paracellular barrier function. Development 131, 4931–4942. 10.1242/dev.01372 15459097

[B47] Farquhar M. G. Palade G. E. (1963). Junctional complexes in various epithelia. J. Cell Biol. 17, 375–412. 10.1083/jcb.17.2.375 13944428 PMC2106201

[B48] Farquhar M. G. Palade G. E. (1965). Cell junctions in amphibian skin. J. Cell Biol. 26, 263–291. 10.1083/jcb.26.1.263 5859021 PMC2106698

[B49] Featherstone D. E. (2011). Glial solute carrier transporters in Drosophila and mice. Glia 59, 1351–1363. 10.1002/glia.21085 21732427

[B51] Fehon R. G. Dawson I. A. Artavanis-Tsakonas S. (1994). A Drosophila homologue of membrane-skeleton protein 4.1 is associated with septate junctions and is encoded by the coracle gene. Development 120, 545–557. 8162854 10.1242/dev.120.3.545

[B52] Fei H. Chow D. M. Chen A. Romero-Calderón R. Ong W. S. Ackerson L. C.. (2010). Mutation of the Drosophila vesicular GABA transporter disrupts visual figure detection. J. Exp. Biol. 213, 1717–1730. 10.1242/jeb.036053 20435823 PMC2861964

[B53] Filippov V. Aimanova K. Gill S. S. (2003). Expression of an Aedes aegypti cation-chloride cotransporter and its Drosophila homologues. Insect Mol. Biol. 12, 319–331. 10.1046/j.1365-2583.2003.00415.x 12864912

[B54] Franzdóttir S. R. Engelen D. Yuva-Aydemir Y. Schmidt I. Aho A. Klämbt C. (2009). Switch in FGF signalling initiates glial differentiation in the Drosophila eye. Nature 460, 758–761. 10.1038/nature08167 19597479

[B55] Freeman M. R. Delrow J. Kim J. Johnson E. Doe C. Q. (2003). Unwrapping glial biology: Gcm target genes regulating glial development, diversification, and function. Neuron 38, 567–580. 10.1016/S0896-6273(03)00289-7 12765609

[B56] Friedman S. (1978). Trehalose regulation, one aspect of metabolic homeostasis. Ann. Rev. Entomol 23, 389–407 10.1146/annurev.en.23.010178.002133

[B57] Fünfschilling U. Supplie L. M. Mahad D. Boretius S. Saab A. S. Edgar J.. (2012). Glycolytic oligodendrocytes maintain myelin and long-term axonal integrity. Nature 485, 517–521. 10.1038/nature11007 22622581 PMC3613737

[B58] Furuse M. Izumi Y. Oda Y. Higashi T. Iwamoto N. (2014). Molecular organization of tricellular tight junctions. Tissue Barriers 2, e28960. 10.4161/tisb.28960 25097825 PMC4117683

[B59] Gaete P. S. Lillo M. A. Figueroa X. F. (2014). Functional role of connexins and pannexins in the interaction between vascular and nervous system. J. Cell. Physiol. 229, 1336–1345. 10.1002/jcp.24563 24446239

[B60] Genova J. L. Fehon R. G. (2003). Neuroglian, Gliotactin, and the Na^+^/K^+^ ATPase are essential for septate junction function in Drosophila. J. Cell Biol. 161, 979–989. 10.1083/jcb.200212054 12782686 PMC2172966

[B61] Giesen K. Hummel T. Stollewerk A. Harrison S. Travers A. Klämbt C. (1997). Glial development in the Drosophila CNS requires concomitant activation of glial and repression of neuronal differentiation genes. Development 124, 2307–2316. 9199357 10.1242/dev.124.12.2307

[B62] Goberdhan D. C. I. Meredith D. Boyd C. A. R. Wilson C. (2005). PAT-related amino acid transporters regulate growth via a novel mechanism that does not require bulk transport of amino acids. Development 132, 2365–2375. 10.1242/dev.01821 15843412

[B64] Graf F. Noirot-Timothée C. Noirot C. (1982). The specialization of septate junctions in regions of tricellular junctions. I. Smooth septate junctions (=continuous junctions). J. Ultrastruct. Res. 78, 136–151. 10.1016/S0022-5320(82)80019-1 7086932

[B65] Grosjean Y. Grillet M. Augustin H. Ferveur J.-F. Featherstone D. E. (2008). A glial amino-acid transporter controls synapse strength and courtship in Drosophila. Nat. Neurosci. 11, 54–61. 10.1038/nn2019 18066061 PMC2196133

[B66] Guan Z. Saraswati S. Adolfsen B. Littleton J. T. (2005). Genome-wide transcriptional changes associated with enhanced activity in the Drosophila nervous system. Neuron 48, 91–107. 10.1016/j.neuron.2005.08.036 16202711

[B67] Hall S. Bone C. Oshima K. Zhang L. McGraw M. Lucas B.. (2014). Macroglobulin complement-related encodes a protein required for septate junction organization and paracellular barrier function in Drosophila. Development 141, 889–898. 10.1242/dev.102152 24496625 PMC3912832

[B68] Harris J. J. Jolivet R. Attwell D. (2012). Synaptic energy use and supply. Neuron 75, 762–777. 10.1016/j.neuron.2012.08.019 22958818

[B69] Hartenstein V. (2011). Morphological diversity and development of glia in Drosophila. Glia 59, 1237–1252. 10.1002/glia.21162 21438012 PMC3950653

[B70] Hatan M. Shinder V. Israeli D. Schnorrer F. Volk T. (2011). The Drosophila blood brain barrier is maintained by GPCR-dependent dynamic actin structures. J. Cell Biol. 192, 307–319. 10.1083/jcb.201007095 21242289 PMC3172179

[B71] Hekmat-Scafe D. S. Lundy M. Y. Ranga R. Tanouye M. A. (2006). Mutations in the K+/Cl- cotransporter gene kazachoc (kcc) increase seizure susceptibility in Drosophila. J. Neurosci. 26, 8943–8954. 10.1523/JNEUROSCI.4998-05.2006 16943550 PMC6675325

[B72] Hijazi A. Masson W. Augé B. Waltzer L. Haenlin M. Roch F. (2009). boudin is required for septate junction organisation in Drosophila and codes for a diffusible protein of the Ly6 superfamily. Development 136, 2199–2209. 10.1242/dev.033845 19502482

[B73] Holcroft C. E. Jackson W. D. Lin W.-H. Bassiri K. Baines R. A. Phelan P. (2013). Innexins Ogre and Inx2 are required in glial cells for normal postembryonic development of the Drosophila central nervous system. J. Cell Sci. 126, 3823–3834. 10.1242/jcs.117994 23813964

[B74] Hosoya T. Takizawa K. Nitta K. Hotta Y. (1995). glial cells missing: a binary switch between neuronal and glial determination in Drosophila. Cell 82, 1025–1036. 10.1016/0092-8674(95)90281-3 7553844

[B75] Hoxha V. Lama C. Chang P. L. Saurabh S. Patel N. Olate N.. (2013). Sex-specific signaling in the blood-brain barrier is required for male courtship in Drosophila. PLoS Genet. 9:e1003217. 10.1371/journal.pgen.1003217 23359644 PMC3554526

[B76] Ile K. E. Tripathy R. Goldfinger V. Renault A. D. (2012). Wunen, a Drosophila lipid phosphate phosphatase, is required for septate junction-mediated barrier function. Development 139, 2535–2546. 10.1242/dev.077289 22675212

[B77] Ito K. Urban J. Technau G. M. (1995). Distribution, classification, and development ofDrosophila glial cells in the late embryonic and early larval ventral nerve cord. Roux's Arch. Dev. Biol. 204, 284–307 10.1007/BF02179499 28306125

[B78] Jang C. Lee G. Chung J. (2008). LKB1 induces apical trafficking of Silnoon, a monocarboxylate transporter, in *Drosophila melanogaster*. J. Cell Biol. 183, 11–17. 10.1083/jcb.200807052 18838551 PMC2557035

[B79] Jaspers M. H. J. Nolde K. Behr M. Joo S.-H. Plessmann U. Nikolov M.. (2012). The claudin Megatrachea protein complex. J. Biol. Chem. 287, 36756–36765. 10.1074/jbc.M112.399410 22930751 PMC3481279

[B80] Jenett A. Rubin G. M. Ngo T.-T. B. Shepherd D. Murphy C. Dionne H.. (2012). A GAL4-driver line resource for Drosophila neurobiology. Cell Rep. 2, 991–1001. 10.1016/j.celrep.2012.09.011 23063364 PMC3515021

[B81] Jeuniaux C. (1971). Hemolymph-Arthropoda, in Chemical Zoology, eds Florkin M. Scheer B. T. (New York; London: Academic Press), 64–118.

[B82] Jolivet R. Magistretti P. J. Weber B. (2009). Deciphering neuron-glia compartmentalization in cortical energy metabolism. Front. Neuroenergetics 1:4. 10.3389/neuro.14.004.2009 19636395 PMC2715922

[B83] Jones B. W. Fetter R. D. Tear G. Goodman C. S. (1995). glial cells missing: a genetic switch that controls glial versus neuronal fate. Cell 82, 1013–1023. 10.1016/0092-8674(95)90280-5 7553843

[B84] Kanamori Y. Saito A. Hagiwara-Komoda Y. Tanaka D. Mitsumasu K. Kikuta S.. (2010). The trehalose transporter 1 gene sequence is conserved in insects and encodes proteins with different kinetic properties involved in trehalose import into peripheral tissues. Insect Biochem. Mol. Biol. 40, 30–37. 10.1016/j.ibmb.2009.12.006 20035867

[B85] Kasuya J. Kaas G. A. Kitamoto T. (2009). A putative amino acid transporter of the solute carrier 6 family is upregulated by lithium and is required for resistance to lithium toxicity in Drosophila. Neuroscience 163, 825–837. 10.1016/j.neuroscience.2009.07.027 19619614 PMC2746873

[B86] Kaufmann N. Mathai J. C. Hill W. G. Dow J. A. T. Zeidel M. L. Brodsky J. L. (2005). Developmental expression and biophysical characterization of a *Drosophila melanogaster* aquaporin. Am. J. Physiol. Cell Physiol. 289, C397–C407. 10.1152/ajpcell.00612.2004 15800049

[B87] Kellett G. L. Brot-Laroche E. Mace O. J. Leturque A. (2008). Sugar absorption in the intestine: the role of GLUT2. Annu. Rev. Nutr. 28, 35–54. 10.1146/annurev.nutr.28.061807.155518 18393659

[B88] Kikawada T. Saito A. Kanamori Y. Nakahara Y. Iwata K.-I. Tanaka D.. (2007). Trehalose transporter 1, a facilitated and high-capacity trehalose transporter, allows exogenous trehalose uptake into cells. Proc. Natl. Acad. Sci. U.S.A. 104, 11585–11590. 10.1073/pnas.0702538104 17606922 PMC1905927

[B89] Klaes A. Menne T. Stollewerk A. Scholz H. Klämbt C. (1994). The Ets transcription factors encoded by the Drosophila gene pointed direct glial cell differentiation in the embryonic CNS. Cell 78, 149–160. 10.1016/0092-8674(94)90581-9 8033206

[B90] Landgraf M. Bossing T. Technau G. M. Bate M. (1997). The origin, location, and projections of the embryonic abdominal motorneurons of Drosophila. J. Neurosci. 17, 9642–9655. 9391019 10.1523/JNEUROSCI.17-24-09642.1997 PMC6573408

[B91] Lane N. J. (1991). Morphology of glial blood-brain barriers. Ann. N.Y. Acad. Sci. 633, 348–362. 10.1111/j.1749-6632.1991.tb15626.x 1789558

[B92] Lane N. J. Abbott N. J. (1992). Freeze-fracture evidence for a novel restricting junction at the blood-brain barrier of the cuttlefish *Sepia officinalis*. J. Neurocytol. 21, 295–303. 10.1007/BF01224762 1588348

[B93] Lane N. J. Swales L. S. (1979). Intercellular junctions and the development of the blood-brain barrier in *Manduca sexta*. Brain Res. 168, 227–245. 10.1016/0006-8993(79)90166-5 445142

[B94] Laprise P. Lau K. M. Harris K. P. Silva-Gagliardi N. F. Paul S. M. Beronja S.. (2009). Yurt, Coracle, Neurexin IV and the Na+,K+-ATPase form a novel group of epithelial polarity proteins. Nature 459, 1141–1145. 10.1038/nature08067 19553998

[B95] Laridon B. Callaerts P. Norga K. (2008). Embryonic expression patterns of Drosophila ACS family genes related to the human sialin gene. Gene Expr. Patterns 8, 275–283. 10.1016/j.gep.2007.12.003 18255354

[B96] Laughlin S. B. de Ruyter van Steveninck R. R. Anderson J. C. (1998). The metabolic cost of neural information. Nat. Neurosci. 1, 36–41. 10.1038/236 10195106

[B97] Lazareva A. A. Roman G. Mattox W. Hardin P. E. Dauwalder B. (2007). A role for the adult fat body in Drosophila male courtship behavior. PLoS Genet. 3:e16. 10.1371/journal.pgen.0030016 17257054 PMC1781494

[B98] Lee G. Park J. H. (2004). Hemolymph sugar homeostasis and starvation-induced hyperactivity affected by genetic manipulations of the adipokinetic hormone-encoding gene in *Drosophila melanogaster*. Genetics 167, 311–323. 10.1534/genetics.167.1.311 15166157 PMC1470856

[B99] Lee Y. Morrison B. M. Li Y. Lengacher S. Farah M. H. Hoffman P. N.. (2012). Oligodendroglia metabolically support axons and contribute to neurodegeneration. Nature 487, 443–448. 10.1038/nature11314 22801498 PMC3408792

[B100] Leino R. L. Gerhart D. Z. van Bueren A. M. McCall A. L. Drewes L. R. (1997). Ultrastructural localization of GLUT 1 and GLUT 3 glucose transporters in rat brain. J. Neurosci. Res. 49, 617–626. 9302083 10.1002/(SICI)1097-4547(19970901)49:5<617::AID-JNR12>3.0.CO;2-S

[B101] Leiserson W. M. Forbush B. Keshishian H. (2011). Drosophila glia use a conserved cotransporter mechanism to regulate extracellular volume. Glia 59, 320–332. 10.1002/glia.21103 21125654 PMC3005002

[B102] Leiserson W. M. Harkins E. W. Keshishian H. (2000). Fray, a Drosophila serine/threonine kinase homologous to mammalian PASK, is required for axonal ensheathment. Neuron 28, 793–806. 10.1016/S0896-6273(00)00154-9 11163267

[B103] Leybaert L. (2005). Neurobarrier coupling in the brain: a partner of neurovascular and neurometabolic coupling? J. Cereb. Blood Flow Metab. 25, 2–16. 10.1038/sj.jcbfm.9600001 15678108

[B104] Li H.-H. Kroll J. R. Lennox S. M. Ogundeyi O. Jeter J. Depasquale G.. (2014). A GAL4 driver resource for developmental and behavioral studies on the larval CNS of Drosophila. Cell Rep. 8, 897–908. 10.1016/j.celrep.2014.06.065 25088417

[B105] Llimargas M. Strigini M. Katidou M. Karagogeos D. Casanova J. (2004). Lachesin is a component of a septate junction-based mechanism that controls tube size and epithelial integrity in the Drosophila tracheal system. Development 131, 181–190. 10.1242/dev.00917 14681183

[B106] Lovick J. K. Ngo K. T. Omoto J. J. Wong D. C. Nguyen J. D. Hartenstein V. (2013). Postembryonic lineages of the Drosophila brain: I. Development of the lineage-associated fiber tracts. Dev. Biol. 384, 228–257. 10.1016/j.ydbio.2013.07.008 23880429 PMC3886848

[B107] MacMillan H. A. Hughson B. N. (2014). A high-throughput method of hemolymph extraction from adult Drosophila without anesthesia. J. Insect Physiol. 63, 27–31. 10.1016/j.jinsphys.2014.02.005 24561358

[B108] Mayer F. Mayer N. Chinn L. Pinsonneault R. L. Kroetz D. Bainton R. J. (2009). Evolutionary conservation of vertebrate blood-brain barrier chemoprotective mechanisms in Drosophila. J. Neurosci. 29, 3538–3550. 10.1523/JNEUROSCI.5564-08.2009 19295159 PMC3040577

[B109] Melom J. E. Littleton J. T. (2013). Mutation of a NCKX eliminates glial microdomain calcium oscillations and enhances seizure susceptibility. J. Neurosci. 33, 1169–1178. 10.1523/JNEUROSCI.3920-12.2013 23325253 PMC3600868

[B110] Miller M. M. Popova L. B. Meleshkevitch E. A. Tran P. V. Boudko D. Y. (2008). The invertebrate B(0) system transporter, D. melanogaster NAT1, has unique d-amino acid affinity and mediates gut and brain functions. Insect Biochem. Mol. Biol. 38, 923–931. 10.1016/j.ibmb.2008.07.005 18718864 PMC2676678

[B111] Miyamoto T. Slone J. Song X. Amrein H. (2012). A fructose receptor functions as a nutrient sensor in the Drosophila brain. Cell 151, 1113–1125. 10.1016/j.cell.2012.10.024 23178127 PMC3509419

[B112] Moussian B. Tång E. Tonning A. Helms S. Schwarz H. Nüsslein-Volhard C.. (2006). Drosophila Knickkopf and Retroactive are needed for epithelial tube growth and cuticle differentiation through their specific requirement for chitin filament organization. Development 133, 163–171. 10.1242/dev.02177 16339194

[B113] Mueckler M. Thorens B. (2013). The SLC2 (GLUT) family of membrane transporters. Mol. Aspects Med. 34, 121–138. 10.1016/j.mam.2012.07.001 23506862 PMC4104978

[B114] Nagelhus E. A. Ottersen O. P. (2013). Physiological roles of aquaporin-4 in brain. Physiol. Rev. 93, 1543–1562. 10.1152/physrev.00011.2013 24137016 PMC3858210

[B115] Naikkhwah W. O'Donnell M. J. (2011). Salt stress alters fluid and ion transport by Malpighian tubules of *Drosophila melanogaster*: evidence for phenotypic plasticity. J. Exp. Biol. 214, 3443–3454. 10.1242/jeb.057828 21957108

[B116] Narasimha M. Uv A. Krejci A. Brown N. H. Bray S. J. (2008). Grainy head promotes expression of septate junction proteins and influences epithelial morphogenesis. J. Cell Sci. 121, 747–752. 10.1242/jcs.019422 18303052

[B117] Nässel D. R. Kubrak O. I. Liu Y. Luo J. Lushchak O. V. (2013). Factors that regulate insulin producing cells and their output in Drosophila. Front. Physiol. 4:252. 10.3389/fphys.2013.00252 24062693 PMC3775311

[B118] Nelson K. S. Furuse M. Beitel G. J. (2010). The Drosophila Claudin Kune-kune is required for septate junction organization and tracheal tube size control. Genetics 185, 831–839. 10.1534/genetics.110.114959 20407131 PMC2907205

[B119] Nilton A. Oshima K. Zare F. Byri S. Nannmark U. Nyberg K. G.. (2010). Crooked, coiled and crimpled are three Ly6-like proteins required for proper localization of septate junction components. Development 137, 2427–2437. 10.1242/dev.052605 20570942 PMC2889608

[B120] Noirot-Timothée C. Graf F. Noirot C. (1982). The specialization of septate junctions in regions of tricellular junctions. II. Pleated septate junctions. J. Ultrastruct. Res. 78, 152–165. 10.1016/S0022-5320(82)80020-8 7086933

[B122] Oda Y. Otani T. Ikenouchi J. Furuse M. (2014). Tricellulin regulates junctional tension of epithelial cells at tricellular contacts via Cdc42. J. Cell Sci. [Epub ahead of print]. 10.1242/jcs.150607 25097232

[B123] Oshima K. Fehon R. G. (2011). Analysis of protein dynamics within the septate junction reveals a highly stable core protein complex that does not include the basolateral polarity protein Discs large. J. Cell Sci. 124, 2861–2871. 10.1242/jcs.087700 21807950 PMC3148133

[B124] Padash-Barmchi M. Browne K. Sturgeon K. Jusiak B. Auld V. J. (2010). Control of Gliotactin localization and levels by tyrosine phosphorylation and endocytosis is necessary for survival of polarized epithelia. J. Cell Sci. 123, 4052–4062. 10.1242/jcs.066605 21045109

[B126] Palanker L. Tennessen J. M. Lam G. Thummel C. S. (2009). Drosophila HNF4 regulates lipid mobilization and β-oxidation. Cell Metab. 9, 228–239. 10.1016/j.cmet.2009.01.009 19254568 PMC2673486

[B127] Palm W. Sampaio J. L. Brankatschk M. Carvalho M. Mahmoud A. Shevchenko A.. (2012). Lipoproteins in *Drosophila melanogaster*–assembly, function, and influence on tissue lipid composition. PLoS Genet. 8:e1002828. 10.1371/journal.pgen.1002828 22844248 PMC3406001

[B128] Papadopoulos M. C. Verkman A. S. (2013). Aquaporin water channels in the nervous system. Nat. Rev. Neurosci. 14, 265–277. 10.1038/nrn3468 23481483 PMC3732112

[B129] Park D. HadŽiæ T. Yin P. Rusch J. Abruzzi K. Rosbash M.. (2011). Molecular organization of Drosophila neuroendocrine cells by dimmed. Curr. Biol. 21, 1515–1524. 10.1016/j.cub.2011.08.015 21885285 PMC3184372

[B130] Pasco M. Y. Léopold P. (2012). High sugar-induced insulin resistance in Drosophila relies on the lipocalin Neural Lazarillo. PLoS ONE 7:e36583. 10.1371/journal.pone.0036583 22567167 PMC3342234

[B131] Paul S. M. Palladino M. J. Beitel G. J. (2007). A pump-independent function of the Na,K-ATPase is required for epithelial junction function and tracheal tube-size control. Development 134, 147–155. 10.1242/dev.02710 17164420 PMC1955469

[B132] Pellerin L. Magistretti P. J. (1994). Glutamate uptake into astrocytes stimulates aerobic glycolysis: a mechanism coupling neuronal activity to glucose utilization. Proc. Natl. Acad. Sci. U.S.A. 91, 10625–10629. 10.1073/pnas.91.22.10625 7938003 PMC45074

[B133] Pellerin L. Magistretti P. J. (2012). Sweet sixteen for ANLS. J. Cereb. Blood Flow Metab. 32, 1152–1166. 10.1038/jcbfm.2011.149 22027938 PMC3390819

[B134] Pereanu W. Shy D. Hartenstein V. (2005). Morphogenesis and proliferation of the larval brain glia in Drosophila. Dev. Biol. 283, 191–203. 10.1016/j.ydbio.2005.04.024 15907832

[B136] Rajan A. Perrimon N. (2012). Drosophila cytokine unpaired 2 regulates physiological homeostasis by remotely controlling insulin secretion. Cell 151, 123–137. 10.1016/j.cell.2012.08.019 23021220 PMC3475207

[B138] Rao Y. Jan L. Y. Jan Y. N. (1990). Similarity of the product of the Drosophila neurogenic gene big brain to transmembrane channel proteins. Nature 345, 163–167. 10.1038/345163a0 1692392

[B139] Reynolds B. Roversi P. Laynes R. Kazi S. Boyd C. A. R. Goberdhan D. C. I. (2009). Drosophila expresses a CD98 transporter with an evolutionarily conserved structure and amino acid-transport properties. Biochem. J. 420, 363–372. 10.1042/BJ20082198 19335336 PMC2685896

[B140] Rickert C. Kunz T. Harris K.-L. Whitington P. M. Technau G. M. (2011). Morphological characterization of the entire interneuron population reveals principles of neuromere organization in the ventral nerve cord of Drosophila. J. Neurosci. 31, 15870–15883. 10.1523/JNEUROSCI.4009-11.2011 22049430 PMC6623031

[B141] Rival T. Soustelle L. Strambi C. Besson M.-T. Iché M. Birman S. (2004). Decreasing glutamate buffering capacity triggers oxidative stress and neuropil degeneration in the Drosophila brain. Curr. Biol. 14, 599–605. 10.1016/j.cub.2004.03.039 15062101

[B142] Romero-Calderón R. Krantz D. E. (2006). Transport of polyamines in Drosophila S2 cells: kinetics, pharmacology and dependence on the plasma membrane proton gradient. Biochem. J. 393, 583. 10.1042/BJ20050981 16248856 PMC1360709

[B143] Romero-Calderón R. Uhlenbrock G. Borycz J. Simon A. F. Grygoruk A. Yee S. K.. (2008). A glial variant of the vesicular monoamine transporter is required to store histamine in the drosophila visual system. PLoS Genet. 4:e1000245. 10.1371/journal.pgen.1000245 18989452 PMC2570955

[B144] Rothstein J. D. Martin L. Levey A. I. Dykes-Hoberg M. Jin L. Wu D.. (1994). Localization of neuronal and glial glutamate transporters. Neuron 13, 713–725. 10.1016/0896-6273(94)90038-8 7917301

[B145] Rouach N. Koulakoff A. Abudara V. Willecke K. Giaume C. (2008). Astroglial metabolic networks sustain hippocampal synaptic transmission. Science 322, 1551–1555. 10.1126/science.1164022 19056987

[B146] Saito M. Awasaki T. Hama C. (2002). Genetic analyses of essential genes in cytological region 61D1-2 to 61F1-2 of *Drosophila melanogaster*. Mol. Genet. Genomics 268, 446–454. 10.1007/s00438-002-0770-6 12471442

[B147] Schmid A. Chiba A. Doe C. Q. (1999). Clonal analysis of Drosophila embryonic neuroblasts: neural cell types, axon projections and muscle targets. Development 126, 4653–4689. 10518486 10.1242/dev.126.21.4653

[B148] Schmidt I. Franzdóttir S. R. Edenfeld G. Rodrigues F. Zierau A. Klämbt C. (2011). Transcriptional regulation of peripheral glial cell differentiation in the embryonic nervous system of Drosophila. Glia 59, 1264–1272. 10.1002/glia.21123 21213301

[B149] Schubiger M. Feng Y. Fambrough D. M. Palka J. (1994). A mutation of the Drosophila sodium pump alpha subunit gene results in bang-sensitive paralysis. Neuron 12, 373–381. 10.1016/0896-6273(94)90278-X 8110464

[B150] Schulte J. Tepass U. Auld V. J. (2003). Gliotactin, a novel marker of tricellular junctions, is necessary for septate junction development in Drosophila. J. Cell Biol. 161, 991–1000. 10.1083/jcb.200303192 12782681 PMC2172969

[B151] Schwabe T. Bainton R. J. Fetter R. D. Heberlein U. Gaul U. (2005). GPCR signaling is required for blood-brain barrier formation in drosophila. Cell 123, 133–144. 10.1016/j.cell.2005.08.037 16213218

[B152] Segawa H. Kawakita M. Ishida N. (2002). Human and Drosophila UDP-galactose transporters transport UDP-N-acetylgalactosamine in addition to UDP-galactose. Eur. J. Biochem. 269, 128–138. 10.1046/j.0014-2956.2001.02632.x 11784306

[B153] Sekine S. U. Haraguchi S. Chao K. Kato T. Luo L. Miura M.. (2013). Meigo governs dendrite targeting specificity by modulating Ephrin level and N-glycosylation. Nat. Neurosci. 16, 683–691. 10.1038/nn.3389 23624514 PMC4779446

[B154] Shandala T. Takizawa K. Saint R. (2003). The dead ringer/retained transcriptional regulatory gene is required for positioning of the longitudinal glia in the Drosophila embryonic CNS. Development 130, 1505–1513. 10.1242/dev.00377 12620977

[B155] Shim J. Gururaja-Rao S. Banerjee U. (2013). Nutritional regulation of stem and progenitor cells in Drosophila. Development 140, 4647–4656. 10.1242/dev.079087 24255094 PMC3833425

[B156] Shim M. S. Kim J. Y. Lee K. H. Jung H. K. Carlson B. A. Xu X. M.. (2011). l(2)01810 is a novel type of glutamate transporter that is responsible for megamitochondrial formation. Biochem. J. 439, 277–286. 10.1042/BJ20110582 21728998 PMC3460806

[B157] Silies M. Yuva Y. Engelen D. Aho A. Stork T. Klämbt C. (2007). Glial cell migration in the eye disc. J. Neurosci. 27, 13130–13139. 10.1523/JNEUROSCI.3583-07.2007 18045907 PMC6673417

[B158] Smith Q. R. (2000). Transport of glutamate and other amino acids at the blood-brain barrier. J. Nutr. 130, 1016S–1022S. 10736373 10.1093/jn/130.4.1016S

[B159] Sousa-Nunes R. Yee L. L. Gould A. P. (2011). Fat cells reactivate quiescent neuroblasts via TOR and glial insulin relays in Drosophila. Nature 471, 508–512. 10.1038/nature09867 21346761 PMC3146047

[B160] Soustelle L. Besson M.-T. Rival T. Birman S. (2002). Terminal glial differentiation involves regulated expression of the excitatory amino acid transporters in the Drosophila embryonic CNS. Dev. Biol. 248, 294–306. 10.1006/dbio.2002.0742 12167405

[B161] Spéder P. Brand A. H. (2014). Gap junction proteins in the blood-brain barrier control nutrient-dependent reactivation of drosophila neural stem cells. Dev. Cell. 30, 309–321. 10.1016/j.devcel.2014.05.021 25065772 PMC4139190

[B183] Stacey S. M. Muraro N. I. Peco E. Labbé A. Thomas G. B. Baines R. A.. (2010). Drosophila glial glutamate transporter Eaat1 is regulated by fringe-mediated notch signaling and is essential for larval locomotion. J. Neurosci. 30, 14446–14457. 10.1523/JNEUROSCI.1021-10.2010 20980602 PMC6634815

[B162] Stork T. Bernardos R. Freeman M. R. (2012). Analysis of glial cell development and function in Drosophila. Cold Spring Harb. Protoc. 2012, 1–17. 10.1101/pdb.top067587 22194269 PMC5193132

[B163] Stork T. Engelen D. Krudewig A. Silies M. Bainton R. J. Klämbt C. (2008). Organization and function of the blood-brain barrier in Drosophila. J. Neurosci. 28, 587–597. 10.1523/JNEUROSCI.4367-07.2008 18199760 PMC6670337

[B164] Stork T. Sheehan A. Tasdemir-Yilmaz O. E. Freeman M. R. (2014). Neuron-glia interactions through the heartless FGF receptor signaling pathway mediate morphogenesis of Drosophila astrocytes. Neuron 83, 388–403. 10.1016/j.neuron.2014.06.026 25033182 PMC4124900

[B165] Sun Q. Tian E. Turner R. J. Ten Hagen K. G. (2010). Developmental and functional studies of the SLC12 gene family members from *Drosophila melanogaster*. Am. J. Physiol. Cell Physiol. 298, C26–C37. 10.1152/ajpcell.00376.2009 19828839 PMC2806154

[B166] Syed M. H. Krudewig A. Engelen D. Stork T. Klämbt C. (2011). The CD59 family member leaky/coiled is required for the establishment of the blood-brain barrier in Drosophila. J. Neurosci. 31, 7876–7885. 10.1523/JNEUROSCI.0766-11.2011 21613501 PMC6633149

[B167] Tatsumi K. Tsuji S. Miwa H. Morisaku T. Nuriya M. Orihara M.. (2009). Drosophila big brain does not act as a water channel, but mediates cell adhesion. FEBS Lett. 583, 2077–2082. 10.1016/j.febslet.2009.05.035 19467350

[B168] Tepass U. Hartenstein V. (1994). The development of cellular junctions in the Drosophila embryo. Dev. Biol. 161, 563–596. 10.1006/dbio.1994.1054 8314002

[B169] Thimgan M. S. Berg J. S. Stuart A. E. (2006). Comparative sequence analysis and tissue localization of members of the SLC6 family of transporters in adult *Drosophila melanogaster*. J. Exp. Biol. 209, 3383–3404. 10.1242/jeb.02328 16916974

[B170] Thomas G. B. van Meyel D. J. (2007). The glycosyltransferase Fringe promotes delta-notch signaling between neurons and glia, and is required for subtype-specific glial gene expression. Development 134, 591–600. 10.1242/dev.02754 17215308

[B171] Tiklová K. Senti K.-A. Wang S. Gräslund A. Samakovlis C. (2010). Epithelial septate junction assembly relies on melanotransferrin iron binding and endocytosis in Drosophila. Nat. Cell Biol. 12, 1071–1077. 10.1038/ncb2111 20935638

[B172] Tsacopoulos M. Veuthey A. L. Saravelos S. G. Perrottet P. Tsoupras G. (1994). Glial cells transform glucose to alanine, which fuels the neurons in the honeybee retina. J. Neurosci. 14, 1339–1351. 8120629 10.1523/JNEUROSCI.14-03-01339.1994 PMC6577565

[B173] Uldry M. Thorens B. (2004). The SLC2 family of facilitated hexose and polyol transporters. Pflugers Arch. 447, 480–489. 10.1007/s00424-003-1085-0 12750891

[B174] Unhavaithaya Y. Orr-Weaver T. L. (2012). Polyploidization of glia in neural development links tissue growth to blood-brain barrier integrity. Genes Dev. 26, 31–36. 10.1101/gad.177436.111 22215808 PMC3258963

[B175] Urbach R. Technau G. M. (2003). Molecular markers for identified neuroblasts in the developing brain of Drosophila. Development 130, 3621–3637. 10.1242/dev.00533 12835380

[B176] Venken K. J. T. Simpson J. H. Bellen H. J. (2011). Genetic manipulation of genes and cells in the nervous system of the fruit fly. Neuron 72, 202–230. 10.1016/j.neuron.2011.09.021 22017985 PMC3232021

[B177] Vincent S. Vonesch J. L. Giangrande A. (1996). Glide directs glial fate commitment and cell fate switch between neurones and glia. Development 122, 131–139. 8565824 10.1242/dev.122.1.131

[B178] Wheeler S. R. Kearney J. B. Guardiola A. R. Crews S. T. (2006). Single-cell mapping of neural and glial gene expression in the developing Drosophila CNS midline cells. Dev. Biol. 294, 509–524. 10.1016/j.ydbio.2006.03.016 16631157 PMC2718739

[B179] Woods D. F. Hough C. Peel D. Callaini G. Bryant P. J. (1996). Dlg protein is required for junction structure, cell polarity, and proliferation control in Drosophila epithelia. J. Cell Biol. 134, 1469–1482. 10.1083/jcb.134.6.1469 8830775 PMC2120992

[B180] Wu V. M. Beitel G. J. (2004). A junctional problem of apical proportions: epithelial tube-size control by septate junctions in the Drosophila tracheal system. Curr. Opin. Cell Biol. 16, 493–499. 10.1016/j.ceb.2004.07.008 15363798

[B181] Wu V. M. Yu M. H. Paik R. Banerjee S. Liang Z. Paul S. M.. (2007). Drosophila Varicose, a member of a new subgroup of basolateral MAGUKs, is required for septate junctions and tracheal morphogenesis. Development 134, 999–1009. 10.1242/dev.02785 17267446 PMC1955473

[B182] Wyatt G. R. Kalf G. F. (1957). The chemistry of insect hemolymph II. Trehalose and other carbohydrates. J. Gen. Physiol. 40, 833–847. 10.1085/jgp.40.6.833 13439163 PMC2147581

